# Motoneuronal inflammasome activation triggers excessive neuroinflammation and impedes regeneration after sciatic nerve injury

**DOI:** 10.1186/s12974-022-02427-9

**Published:** 2022-03-19

**Authors:** Kinga Molnár, Bernát Nógrádi, Rebeka Kristóf, Ádám Mészáros, Krisztián Pajer, László Siklós, Antal Nógrádi, Imola Wilhelm, István A. Krizbai

**Affiliations:** 1 Institute of Biophysics, Biological Research Centre, Eötvös Loránd Research Network (ELKH), Temesvári krt. 62, 6726 Szeged, Hungary; 2 Theoretical Medicine Doctoral School, University of Szeged, Szeged, Hungary; 3 Doctoral School of Biology, University of Szeged, Szeged, Hungary; 4 Department of Anatomy, Histology and Embryology, University of Szeged, Szeged, Hungary; 5 Institute of Life Sciences, Vasile Goldiş Western University of Arad, Arad, Romania; 6 Present Address: Department of Neurology, University of Szeged, Szeged, Hungary

**Keywords:** Axotomy, Extracellular ATP, IL-1β, Inflammasome, Motoneuron, NLRP3, Regeneration, Reinnervation, Sciatic nerve injury

## Abstract

**Background:**

Peripheral nerve injuries are accompanied by inflammatory reactions, over-activation of which may hinder recovery. Among pro-inflammatory pathways, inflammasomes are one of the most potent, leading to release of active IL-1β. Our aim was to understand how inflammasomes participate in central inflammatory reactions accompanying peripheral nerve injury.

**Methods:**

After axotomy of the sciatic nerve, priming and activation of the NLRP3 inflammasome was examined in cells of the spinal cord. Regeneration of the nerve was evaluated after coaptation using sciatic functional index measurements and retrograde tracing.

**Results:**

In the first 3 days after the injury, elements of the NLRP3 inflammasome were markedly upregulated in the L4–L5 segments of the spinal cord, followed by assembly of the inflammasome and secretion of active IL-1β. Although glial cells are traditionally viewed as initiators of neuroinflammation, in this acute phase of inflammation, inflammasome activation was found exclusively in affected motoneurons of the ventral horn in our model. This process was significantly inhibited by 5-BDBD, a P2X4 receptor inhibitor and MCC950, a potent NLRP3 inhibitor. Although at later time points the NLRP3 protein was upregulated in microglia too, no signs of inflammasome activation were detected in these cells. Inhibition of inflammasome activation in motoneurons in the first days after nerve injury hindered development of microgliosis in the spinal cord. Moreover, P2X4 or inflammasome inhibition in the acute phase significantly enhanced nerve regeneration on both the morphological and the functional levels.

**Conclusions:**

Our results indicate that the central reaction initiated by sciatic nerve injury starts with inflammasome activation in motoneurons of the ventral horn, which triggers a complex inflammatory reaction and activation of microglia. Inhibition of neuronal inflammasome activation not only leads to a significant reduction of microgliosis, but has a beneficial effect on the recovery as well.

**Supplementary Information:**

The online version contains supplementary material available at 10.1186/s12974-022-02427-9.

## Background

Neuroinflammation is a major determinant of acute neuronal injury and neurodegeneration. Although injury-induced inflammatory changes may have both beneficial and detrimental aspects [1, 2], over-activation of pro-inflammatory pathways is known to negatively influence regenerative outcome [3, 4]. As an initial step of the activation of immune mechanisms, elements of the innate immune system rapidly react to the accumulation of damage-associated molecular patterns (DAMPs), which are endogenous danger signals that are discharged to the extracellular space in response to injury [5]. DAMPs activate pattern recognition receptors (PRRs), including nucleotide-binding oligomerization domain/NOD-, leucine-rich repeat/LRR- and pyrin domain-containing proteins (NLRPs), such as NLRP3, NLRP1 or NLRP6, or other proteins, like absent in melanoma 2 (AIM2). This leads to the assembly of inflammasomes [6] through PRR oligomerization and recruitment of the adaptor protein apoptosis-associated speck-like protein containing a caspase-recruitment domain (ASC), which in turn binds and induces autoactivation of caspase-1. As a final result of inflammasome activation, pro-inflammatory cytokines interleukin-1β (IL-1β) and IL-18 are processed to their active form and secreted into the extracellular space [7].

Among the wide range of DAMPs, extracellular adenosine triphosphate (ATP) is a multi-target danger signal, which increases neuronal susceptibility to damage and induces inflammatory changes mostly through the P2X purinergic receptor subfamily [8]. Extracellular ATP is one of the stimuli, which potently activates the NLRP3 inflammasome [7]. This process takes place in two steps, first the priming, which means increased expression of inflammasome components and of IL-1β, and then a second, activator signal is required to induce inflammasome assembly [7]. This latter can be the result of the binding of extracellular ATP to a purinergic receptor. The extracellular ATP-mediated P2X4 receptor activation has been documented to induce NLRP3 inflammasome activation in different conditions, such as diabetic nephropathy [9], acute kidney injury [10] and spinal cord injury as well [11].

Although NLRP3 activation has been described in different neurodegenerative conditions [12, 13] and observed in various cell types of the central nervous system (CNS), including astrocytes, microglia and neurons [14–17], our understanding on the exact mechanism of action and the possibilities for therapeutical interventions is still limited. Previously, we described the motoneuron-specific upregulation of NLRP3 in the brainstem following hypoglossal nerve axotomy, accompanied by increased levels of mature IL-1β [18]. Here, we aimed at understanding the role of NLRP3 inflammasome activation in sciatic nerve injury. IL-1β, the end-product of the NLRP3 pathway, is able to amplify motoneuronal injury through excitotoxicity [19] and mediation of microglia and macrophage recruitment and activation in the CNS [20]. Furthermore, IL-1β can also act as a positive regulator of NLRP3 priming [21], thus the initial inflammasome activation can further perpetuate over-activation of neuroinflammation. Therefore, this study focuses on the possible involvement of the ATP–P2X4–NLRP3–IL-1β pathway in motoneuronal injury, microgliosis and the potential pro-regenerative capacity of NLRP3 inhibition.

## Methods

### Experimental animal surgeries and treatments

8- to 12-week-old male BALB/c mice (mean body weight 22 ± 3 g) were housed and treated in the animal facility of the Biological Research Centre, Szeged. All procedures were approved by the Ethical Committee for the Protection of Animals in Scientific Research at the Biological Research Centre and the Regional Animal Health and Food Control Station of Csongrád-Csanád County (permit number: XVI./767/2018 and XVI./819/2021) and were performed in accordance with widely accepted standards and Hungarian governmental laws related to animal protection.

The number of animals and different experimental groups are summarized in Table 1.

**Table 1:** Number of animals used in different experimental setups

Methods	Experiments	Subgroups/treatments	Number of animals used
qPCR	Time-dependent changes in the expression of inflammasome-related genes	Intact	3
		Sham	3
		6 h after axotomy	3
		1 day after axotomy	3
		3 days after axotomy	3
		7 days after axotomy	3
		21 days after axotomy	3
	Effect of NLRP3 and P2X4 inhibition	Intact	3
		1 day after ax. + vehicle	3
		1 day after ax. + 5-BDBD	3
		1 day after ax. + MCC950	3
ISH	Cellular localization of IL1B mRNA	1 day after axotomy	3
		1 day after intraspinal injection of LPS + MDP (positive control)	1
IF	Colocalization of inflammasome components	1 day after axotomy	3
		3 days after axotomy	3
		7 days after axotomy	3
	Quantification NLRP3 and Iba1/GFAP colocalization	7 days after ax. + vehicle	3
		7 days after ax. + MCC950	3
	Quantification of axonal regeneration	5 days after ax./coapt. + vehicle	3
		5 days after ax./coapt. + MCC950	3
		5 days after ax./coapt. + intrathecal goat IgG	4
		5 days after ax./coapt. + intrathecal IL-1β neutralization	4
WB	Effect of NLRP3 and P2X4 inhibition	3 days after ax. + vehicle	3
		3 days after ax. + 5-BDBD	3
		3 days after ax. + MCC950	3
IHC	Quantification of microglia and astroglia activation	3 days after axotomy	4
		3 days after ax. + vehicle	4
		3 days after ax. + MCC950	4
		3 days after ax. + intrathecal goat IgG	4
		3 days after ax. + intrathecal IL-1β neutralization	4
		7 days after axotomy	4
		7 days after ax. + vehicle	4
		7 days after ax. + MCC950	4
		7 days after ax. + intrathecal goat IgG	4
		7 days after ax. + intrathecal IL-1β neutralization	4
SFI and retrograde labeling	Quantification of sciatic nerve regeneration	Axotomy and coaptation	5
		Ax./coapt. + vehicle	5
		Ax./coapt. + 5-BDBD	5
		Ax./coapt. + MCC950	5
Total number of animals			135

*Ax.* axotomy, *Ax./coapt.* axotomy and coaptation, *qPCR* real-time polymerase chain reaction, *ISH* in situ hybridization, *IF* immunofluorescence, *WB* western blot, *IHC* immunohistochemistry, *SFI* sciatic functional index

Animals were deeply and reversibly anaesthetized with intraperitoneal injection of Avertin (tribromoethanol, Cat# T48402, Merck-Sigma, St. Louis, MN, USA; 240 mg/kg body weight). In animals, where only the effects of axotomy were tested, the right sciatic nerve was carefully exposed, and a 3-mm-long segment was removed at the mid-thigh level to prevent regeneration. In experimental groups aiming to assay regeneration of sciatic nerve axons, the proximal and distal stumps of the transected nerve were epineurially coapted with two sutures (Daclon, Cat# ON105, USP 10/0, EP 0.2, SMI, St. Vith, Belgium) under an operating microscope (OM-5, Takagi Seico, Tokyo, Japan). The wound was closed with Vetbond™ tissue adhesive (Cat# 1469SB, 3 M, St. Paul, MN, USA).

Operated animals received intraperitoneal injection of 10 mg/kg body weight MCC950 (Cat# inh-mcc, Invivogen, San Diego, CA, USA) 1 h after axotomy or 5 mg/kg body weight 5-BDBD (Cat# 3579, Tocris Bioscience, Bristol, UK) 30 min after injury and then every 24 h up to the third post-operative day. The animals received a single dose or three doses depending on whether they were sacrificed at an earlier or later time point (on day 1 or 3, respectively). Vehicle group received equal amount of dimethyl-sulfoxide (DMSO) (Cat# D2650, Merck-Sigma) in phosphate buffered saline (PBS) intraperitoneally.

For IL-1β neutralization experiments, animals were anaesthetized via 4% (v/v) inhaled isoflurane in oxygen for induction and 1–2% (v/v) for maintenance, from a precision vaporizer (Open Circuit Isoflurane Tabletop System, Stoelting, Dublin, Ireland). Animals with sciatic nerve axotomy received daily intrathecal injections of IL-1β neutralizing antibody (20 µg/kg body weight; Cat# AF-401-NA, RRID:AB_416684, R&D Systems, Minneapolis, MN, USA) diluted in 20 µl of sterile PBS. Intrathecal delivery of the antibody was carried out through the L5–L6 intervertebral space by using a Hamilton syringe with a 30G needle. The successful intrathecal administration was verified by the flick of the tail. First dose was administered 1 h after axotomy. Intrathecal injections were then repeated every 24 h, altogether three (3-day survival) or four doses (5- and 7-day survival). Control group received equal amount of goat IgG (Cat# AB-108-C, RRID:AB_354267, R&D Systems) in equal volume of PBS intrathecally at the same time points.

### RNA isolation and real-time polymerase chain reaction (qPCR)

PCR evaluations were performed using altogether 33 animals. In experiments characterizing the time-dependent changes in gene expression of inflammasome components and mediators, animals were allowed to survive after sciatic nerve axotomy for 6 h, 1, 3, 7 or 21 days. The experimental setup was further supplemented with an absolute control group and a sham-operated group, in which case the sciatic nerve was exposed, but not axotomized. The sham-operated animals were allowed to survive for 1 day following surgery. To evaluate the effect of 5-BDBD and MCC950 treatments on gene expression, animals were separated into 3 groups (vehicle control, 5-BDBD- and MCC950-treated), and were sacrificed 1 day after axotomy.

At the end of the appropriate post-operative time, animals were transcardially perfused with 10 mM PBS (pH = 7.4), then the L4–L5 spinal cord segment was dissected. Spinal cord samples were halved along the rostrocaudal axis, and only the injured half was used for sample preparation. Tissue samples were homogenized in TRIzol reagent (Cat# 15596026, Thermo Fisher Scientific, Waltham, MA, USA) and the total RNA was isolated by using Direct-zol RNA Miniprep Plus kit (Cat# R2071, Zymed Laboratories, Irvine, CA, USA). Maxima First Strand cDNA Synthesis Kit (Cat# K1672, Thermo Fisher Scientific) was used to transcribe RNA into cDNA. Amplification was performed using iTaq™ Universal SYBR® Green Supermix (Cat# 1725122, Bio‐Rad, Hercules, CA, USA) on a Bio-Rad CFX96 Real-Time PCR instrument (RRID:SCR_018064, Bio‐Rad) under the following conditions: 40 cycles of 95 °C for 15 s, 60 °C for 30 s and 72 °C for 30 s using the primers detailed in Table 2.

**Table 2:** Primers used for qPCR

Genes	Forward primers (5’ → 3’)	Reverse primers (5’ → 3’)
*IL1B*	TGCCACCTTTTGACAGTGATG	TGATGTGCTGCTGCGAGATT
*CASP1*	GGGACCCTCAAGTTTTGCC	GACGTGTACGAGTGGTTGTATT
*NLRP3*	GGCGAGACCTCTGGGAAAAA	CTTCAAGGCTGTCCTCCTGG
*NLRP6*	AGCTGAGAACGCTGTGTCG	AACTTGGGAACCCCGAAGC
*AIM2*	GGTGGCGTCAGGAAGTTTTC	GCCGGTCAACAACAGCATTT
*P2X4*	AAAGGTGTGGCTGTGACCAA	TCCAGTCCCAATTCCACTGC
*GAPDH*	GTGAAGGTCGGTGTCAACG	GTGAAGACGCCAGTAGACTC

### In situ hybridization (ISH)

Animals underwent unilateral sciatic nerve axotomy and were transcardially perfused one day later with 10 mM PBS (pH = 7.4), followed by fixation with 4% paraformaldehyde (PFA, Cat# 818715, Merck-Sigma) in 10 mM PBS. Then, the L4–L5 spinal cord segment was dissected. Samples were treated according to the manufacturer’s recommendations (RNAScope 2.5 HD reagent kit-brown, Cat# 322300, Advanced Cell Diagnostics, Milano, Italy). We used Probe-Mm-Il1b (Cat# 316,891, Advanced Cell Diagnostics) to detect IL1B mRNA expression in the spinal cord. Tissue sections were counterstained with 50% Gill’s haematoxylin. As a positive control, the combination of 1 µg/µl lipopolysaccharide (LPS, Cat# L2630, Merck-Sigma) and 0.01 µg/µl muramyl dipeptide (MDP, Cat# G-1055, Bachem, Bubendorf, Switzerland) were injected into the L4-L5 dorsolateral spinal cord with a glass micropipette with a tip of 50 µm diameter in a final volume of 500 nl to induce inflammasome activation, resulting in the elevation of IL1B mRNA expression (Additional file 1: Fig. S1a).

### Methanol–chloroform precipitation of proteins and western blot (WB)

For WB experiments, surgical unilateral axotomy of the sciatic nerve was conducted on 9 animals. Animals were divided into three equal groups (*N* = 3), including vehicle control, 5-BDBD- and MCC950-treated groups. 3 days after the axotomy, animals were irreversibly anaesthetized with Avertin and underwent transcardial perfusion with 10 mM PBS (pH = 7.4). The spinal cord was exposed, removed and placed into 10 mM PBS, then dissected in the following manner: first, the spinal cord was sliced along the mid-sagittal axis and the two sides (injured and control sides) were separated, then the L4–L5 segment was dissected and used further (L4–L5 injured and L4–L5 control). Dorsal root ganglia (DRG) and exiting spinal nerves were carefully removed. Snap-freezing in liquid nitrogen was performed immediately after tissue dissection, then samples were processed in a Potter–Elvehjem homogenizer with a PTFE pestle in 10 mM PBS supplemented with 1 mM Pefabloc® SC (Cat# 11429868001, Merck-Sigma) and 1 mM ethylenediaminetetraacetic acid (EDTA). The homogenizer was thoroughly washed multiple times with distilled water between the samples. Protein concentration was determined by using the bicinchoninic acid assay (BCA, Cat# 23225, Thermo Fisher Scientific).

Equal volume of 100% ice-cold methanol and ¼ volume of chloroform were added to the homogenized samples, vortexed and centrifuged at 13 000 × *g* for 5 min at 4 °C. After phase separation, the aqueous phase was removed, and the protein pellet was washed with methanol. Samples were vortexed and centrifuged again. The supernatant was discarded, and the protein pellet was dried. Pellets were reconstituted in 2 × Laemmli buffer and heated up to 95 °C for 5 min.

Samples were separated using standard denaturing SDS/PAGE and blotted on polyvinylidene difluoride membranes (PVDF; 0.2 μm pore size; Cat# 162–0177, Bio-Rad). After blocking with 3% bovine serum albumin (BSA; Cat# 97061-422, VWR International, Radnor, PA, USA) in Tris-buffered saline with 0.1% Tween-20 (TBS-T), membranes were incubated with primary antibodies (Table 3) overnight at 4 °C. Blots were washed in TBS‐T three times for 10 min, incubated for 1 h in horseradish peroxidase‐conjugated anti‐rabbit IgG, anti‐mouse IgG (Cat# 115–035-003, RRID: AB_10015289, Jackson ImmunoResearch, Cambridgeshire, UK) or anti-goat IgG (Cat# A5420, RRID:AB_258242, Merck-Sigma) secondary antibodies (Table 3) diluted to 1:4000 in TBS‐T, and then washed again in TBS‐T. Immunoreaction was visualized with Clarity Chemiluminescence Substrate (Cat# 1705061, Bio‐Rad) in a ChemiDoc MP System (RRID:SCR_019037, Bio‐Rad). Densitometry analysis was performed with the Image Lab Software, version 5.2 (RRID:SCR_014210, Bio‐Rad).

**Table 3:** Primary and secondary antibodies used for western blot (WB), immunohistochemistry (IHC) and immunofluorescence (IF)

Stainings	Primary antibodies	Secondary antibodies
IL-1β (WB)	Polyclonal goat against IL-1β, 1:500 (Cat# AF-401-NA, RRID:AB_416684, R&D Systems)	HRP-conjugated rabbit anti-goat IgG (H + L), 1:4000 (Cat# A5420, RRID:AB_258242, Merck-Sigma)
β-actin (WB)	Monoclonal mouse against β-actin, 1:10,000 (Cat# A5441, RRID:AB_476744, Merck-Sigma)	HRP-conjugated goat anti-mouse IgG (H + L), 1:4000 (Cat# 115-035-003, RRID: AB_10015289, Jackson ImmunoResearch)
Iba1 (IHC)	Polyclonal rabbit against Iba1, 1:500 (Cat# 019-19741, RRID:AB_839504, FUJIFILM Wako Pure Chemical Corporation, Osaka, Japan)	Biotinylated goat anti-Rabbit IgG Antibody (H + L), 1:500 (Cat# BA-1000, RRID:AB_2313606, Vector Laboratories)
GFAP (IHC)	Polyclonal rabbit against GFAP, 1:500 (Cat# ab16997, RRID:AB_443592, Abcam, Cambridge, UK)	Biotinylated goat anti-rabbit IgG Antibody (H + L), 1:500 (Cat# BA-1000, RRID:AB_2313606, Vector Laboratories)
NLRP3 (IF)	Polyclonal goat against NLRP3, 1:100 (Cat# GTX88190, RRID:AB_10723786 GeneTex, Irvine, CA, USA)	Alexa Fluor® 594 Plus Highly Cross-Adsorbed donkey anti-goat IgG (H + L), 1:500 (Cat# A32758, RRID:AB_2762828, Thermo Fisher Scientific)
ASC (IF)	Monoclonal mouse against ASC, 1:100 (Cat# sc-271054, RRID:AB_10608960, Santa Cruz Biotechnology, Dallas, TX, USA)	Alexa Fluor® 647 Plus Highly Cross-Adsorbed donkey anti-mouse IgG (H + L), 1:500 (Cat# A32787, RRID:AB_2762830, Thermo Fisher Scientific)
	Monoclonal rabbit against ASC, 1:100 (Cat# 67-824, RRID:AB_2799736, Cell Signaling Technology, Danvers, MA, USA)	Alexa Fluor® 488 AffiniPure donkey anti-rabbit IgG (H + L), 1:500 (Cat# 711-545-152, RRID:AB_2313584, Jackson ImmunoResearch)
NeuN (IF)	Polyclonal rabbit against NeuN, 1:4000 (Cat# ABN78, RRID:AB_10807945, Merck Millipore)	Alexa Fluor® 488 AffiniPure donkey anti-rabbit IgG (H + L), 1:500 (Cat# 711-545-152, RRID:AB_2313584, Jackson ImmunoResearch)
ChAT (IF)	Polyclonal rabbit against ChAT, 1:250 (Cat# GTX113164, RRID:AB_1949973, GeneTex)	Alexa Fluor® 488 AffiniPure donkey anti-rabbit IgG (H + L), 1:500 (Cat# 711-545-152, RRID:AB_2313584, Jackson ImmunoResearch)
	Polyclonal goat against ChAT, 1:500 (Cat# AB144, RRID:AB_90650, Merck Millipore)	Alexa Fluor® 488 Cross-Absorbed donkey anti-goat IgG (H + L), 1:500 (Cat# A11055, RRID:AB_2534102, Thermo Fisher Scientific)
GFAP (IF)	Polyclonal rabbit against GFAP, 1:500 (Cat# ab16997, RRID:AB_443592, Abcam)	Alexa Fluor® 488 AffiniPure donkey anti-rabbit IgG (H + L), 1:500 (Cat# 711-545-152, RRID:AB_2313584, Jackson ImmunoResearch)
		Alexa Fluor® 647 AffiniPure donkey anti-rabbit IgG (H + L), 1:500 (Cat# 711-605-152, RRID:AB_2492288, Jackson ImmunoResearch)
Iba1 (IF)	Polyclonal rabbit against Iba1, 1:500 (Cat# 019–19741, RRID:AB_839504, FUJIFILM Wako Pure Chemical Corporation)	Alexa Fluor® 488 AffiniPure donkey anti-rabbit IgG (H + L), 1:500 (Cat# 711-545-152, RRID:AB_2313584, Jackson ImmunoResearch)
TRPV1 (IF)	Polyclonal rabbit against TRPV1, 1:500 (Cat# ACC-030, RRID:AB_2313819, Alomone Labs, Jerusalem, Israel)	Alexa Fluor® 488 AffiniPure donkey anti-rabbit IgG (H + L), 1:500 (Cat# 711-545-152, RRID:AB_2313584, Jackson ImmunoResearch)
p75 (IF)	Monoclonal mouse against p75 NGF receptor, 1:500 (Cat# ab6172, RRID:AB_305340, Abcam)	Alexa Fluor® 647 Plus Highly Cross-Adsorbed donkey anti-mouse IgG (H + L), 1:500 (Cat# A32787, RRID:AB_2762830, Thermo Fisher Scientific)
NEFM (IF)	Monoclonal recombinant rabbit against NEFM, 1:500 (Cat# MA5-32613, RRID:AB_2809890, Thermo Fisher Scientific)	Alexa Fluor® 488 AffiniPure donkey anti-rabbit IgG (H + L), 1:500 (Cat# 711-545-152, RRID:AB_2313584, Jackson ImmunoResearch)

### Immunohistochemistry (IHC) and immunofluorescence (IF) stainings

Axotomized animals were perfused with 10 mM PBS (pH = 7.4), followed by fixation with 4% PFA in 10 mM PBS. The spinal cord, the DRG and the injured nerve segments were exposed and removed, then fixed further overnight in the same fixative at 4 °C. Next day the fixative was removed, and the tissue was cryoprotected in 30% sucrose (Cat# 02200-203-190, Molar Chemicals, Halásztelek, Hungary) dissolved in 10 mM PBS, at least for 1 day at 4 °C. Spinal cord samples were mounted onto a freezing microtome (Reichert‐Jung, Leica Biosystems, Wetzlar, Germany) and 30 µm‐thick sections were cut from the L4–L5 lumbar segment. Sections were kept in 10 mM PBS with 0.05% sodium azide (Cat# S2002, Merck-Sigma) until further processing.

IHC stainings were performed on 30 µm-thick free-floating cryo-sections. For the quantitative evaluation of changes in Iba1 and glial fibrillary acidic protein (GFAP) expression, diaminobenzidine tetrahydrochloride (DAB)-based visualization protocol was performed. Sections were rinsed in 10 mM PBS thrice, then to block the endogenous peroxidase activity, we incubated the tissue in 0.6% hydrogen peroxide in 10 mM PBS containing 0.2% Triton X-100 (Cat# T9284, Merck-Sigma) (TPBS) for 30 min. After washing steps with 10 mM PBS, 2% normal goat serum (Cat# S-1000, RRID:AB_2336615, Vector Laboratories, Burlingame, CA, USA) in TPBS was applied for 60 min to block nonspecific binding sites. Primary antibodies (Table 3) were diluted in blocking solution and sections were incubated overnight at 4 °C on an orbital shaker (50–60 rpm). Sections were extensively washed in 10 mM PBS. This was followed by incubation at room temperature in a biotinylated goat anti-rabbit secondary antibody (Table 3) diluted in blocking solution for 60 min. Sections were rinsed in 10 mM PBS three times, incubated in avidin–biotin complex (Cat# PK-6100, RRID:AB_2336819, Vector Laboratories) diluted to 1:800 in PBS for 60 min at room temperature. After thoroughly washing in 10 mM PBS, reactions were visualized by incubation in 0.05% DAB (Cat# 34001, Thermo Fisher Scientific) with 1.5% NiCl_2_ in 10 mM PBS for 15 min. Finally, sections were washed in 10 mM PBS, rinsed in tridistilled water, mounted with Entellan (Cat# 107961, Merck Millipore, Darmstadt, Germany) and visualized under a brightfield microscope (Eclipse 80i, RRID:SCR_015572, Nikon, Tokyo, Japan).

IF staining protocols were also performed on 30 µm-thick free-floating lumbar spinal cord sections. Beside these samples, 10 µm-thick, longitudinal sciatic nerve and dorsal root ganglia transections were cut with a Leica CM1860 cryostat and used for IF stainings.

First, sections were rinsed as previously described. Afterwards, blocking step was used with 2% normal donkey serum (Cat# 017–000-121, RRID:AB_2337258, Jackson ImmunoResearch) in 10 mM TPBS for 60 min. Primary antibody (Table 3) cocktails in blocking solution were applied for overnight incubation at 4 °C. The next day, sections were rinsed and secondary antibody (Table 3) cocktails were used for 60 min. Finally, sections were washed in 10 mM PBS three times and, where indicated, Hoechst 33342 (Cat# B2261, Merck-Sigma) staining was applied (diluted to 1 μg/ml in 10 mM PBS) to visualize cell nuclei. Sections were mounted on silane-coated glass slides, covered with Fluoromount-G (Cat# 0100–01, Southern Biotech, Birmingham, AL, USA) mounting medium. Secondary antibody staining controls have been carried out for each of the applied secondary antibodies to exclude interference of any associated unspecific staining.

Confocal images were obtained with a Leica TCS SP5 laser scanning microscope (RRID:SCR_020233, Leica Biosystems). Images were taken with a HCX PL APO CS 20 × /0.7 or HCX PL APO lambda blue 63 × /1.4 oil objective in 1024 × 1024 resolution. Multiple images were taken from approx. 20 µm depth of field and merged these into z-projections in Las X Viewer (RRID:SCR_013673, Leica Biosystems) or in FIJI (Fiji is just ImageJ, RRID:SCR_002285, Max Planck Institute of Molecular Cell Biology and Genetics, Dresden, Germany) software. In Results, z-stacks were presented, and in some cases, pseudocolors were used to simplify the visualization of certain stainings. Brightness and contrast were adjusted as needed.

### Quantification of microgliosis and astrogliosis

The quantification method was previously used and described by our laboratory [18, 22], based on an interactive macro for the Image-Pro Plus image analysis software (RRID:SCR_016879, Media Cybernetics, Rockville, MD, USA). Briefly, 8 spinal cord sections for each groups were stained for Iba1 and glial fibrillary acidic protein (GFAP) with DAB-based IHC from each animal (*N* = 4 animals/group). Next, both the control and injured ventral horns on the sections were captured with a Nikon Eclipse 80i microscope equipped with a 2560 × 1920 pixel resolution MicroPublisher 5.0 RTV charge-coupled device camera at 20 × magnification. During the image analysis, a consistent background subtraction algorithm was applied, based on internal controls (contralateral side) in each section, to determine the significantly stained profiles in identical regions at both the operated and contralateral sides of the spinal cord. This resulted in an automated, unbiased evaluation procedure, since the algorithm determined the stained profile values with the same background subtraction for both the injured and contralateral sides on each section. Last, we determined and averaged the algebraic differences between the operated and control sides.

### Quantification of microglial and astroglial localization of NLRP3

30 μm-thick spinal cord sections were stained for NLRP3, Iba1 and GFAP, as previously described. Images of the injured ventral horn were captured with a Leica TCS SP5 laser scanning microscope at 63 × magnification with z-stack image acquisition mode, using standardized confocal and z-stack settings across each section. Images were then processed with FIJI (Fiji is just ImageJ, RRID:SCR_002285, Max Planck Institute of Molecular Cell Biology and Genetics, Dresden, Germany) using a standardized protocol. Pixel counts were determined for Iba1, GFAP, NLRP3-Iba1 colocalization and NLRP3-GFAP colocalization. The number of NLRP3-Iba1 and NLRP3-GFAP colocalizing pixels were normalized to the number of Iba1 or GFAP pixels, respectively, thus we could determine the ratio of colocalizing pixels relative to the increased microglial or astroglial activation. This evaluation was carried out 7 days post-axotomy on vehicle-treated and MCC950-treated animals (*N* = 4 animals/group, 6 sections/each animal).

### Sciatic functional index (SFI) measurements

The SFI is a well-characterized index, widely used to study peripheral nerve regeneration. To assess the regeneration of sciatic nerve injury, SFI measurements were carried out 1 day before the axotomy + nerve coaptation and on post-operative days 3, 7 and then on every seventh day up to 8 weeks. The measurements were carried out on untreated, vehicle-treated (DMSO), 5-BDBD-treated and MCC950-treated animals. Treatments were administered as described earlier. A non-toxic dye was used to register the hind paw print of the animals during the walking track analysis. Since earlier studies found that paw length and toe spread are significant indicators of sciatic function in mouse, we used these parameters and the reworked SFI formula for mouse: $SFI=118.9*\frac{ETS-NTS}{NTS}-51.2*\frac{EPL-NPL}{NPL}-7.5$, where NTS: normal toe spread, NPL: normal paw length, ETS: experimental toe spread, EPL: experimental paw length [23]. At the end-point of the experiments, the same animals were used for Fast blue (FB) tracer experiments.

### Retrograde labeling experiments

Eight weeks after the surgery, animals were deeply anaesthetized as described above. On the operated side, the sciatic nerve was cut 2 mm distally to the coaptation and the proximal stump of the nerve was covered with FB crystals (Cat# 17740-5, Polysciences Europe GmbH, Hirschberg an der Bergstrasse, Germany) [24]. On the intact side, the same surgical procedure was performed. Four days after the application of this fluorescent dye, the animals were re-anaesthetized and were perfused transcardially with 4% PFA in 10 mM PBS. Sample preparation was carried out as described earlier for IF staining.

After sectioning of the spinal cord samples, the following routine was applied for the selection of sections for staining: one section (30 μm-thick) was collected, then the next two consecutive sections were omitted (altogether 60 μm-thick). Then, ChAT IF staining was used to label motoneurons. During the cell counting procedure, the ventral horns of both the control and injured sides were captured (on a Nikon Eclipse 80i microscope) on all of the selected sections. FB-positive cells were only counted if they were ChAT-positive, and the cell nucleus was clearly visible. Thus, we could ensure that each motoneuron was only counted once. The number of FB-positive motoneurons was then summed on each side and a ratio could be calculated (injured/contralateral side).

### Statistical analysis

All statistical analyses were performed with GraphPad Prism (version 8.0.1.244, RRID:SCR_002798, GraphPad Software, San Diego, CA, USA). The type of statistical analysis is indicated in the figure legends. Data are represented as mean ± SEM. In order to determine the number of animals needed, power analysis was carried out with G* Power (RRID:SCR_013726)[25]. All experiments were performed in a blinded manner.

## Results

### Lumbar motoneurons activate the NLRP3 inflammasome in response to sciatic nerve injury

**Fig. 1:**
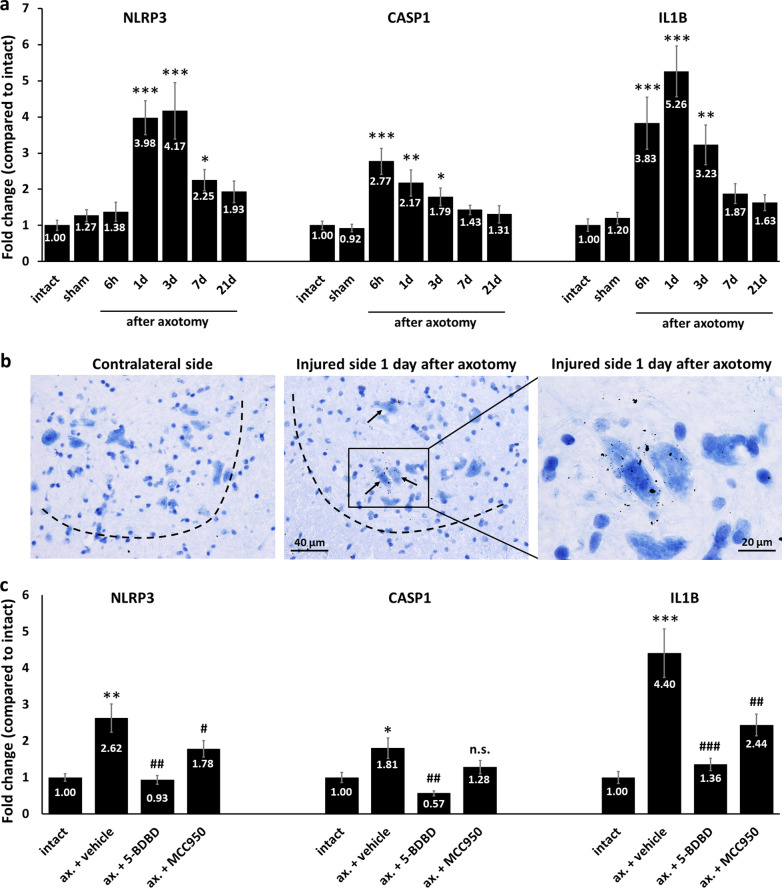
Gene expression changes of inflammasome components in the spinal cord following sciatic nerve axotomy. **a** Changes in the expression of NLRP3, CASP1 and IL1B mRNAs at various time points (6 h, 1 day, 3 days, 7 days and 21 days) after nerve injury, as assessed by qPCR. *N* = 3 animals/group, *p < 0.05, **p < 0.01, ***p < 0.001 (ANOVA with Fisher’s LSD post hoc, compared to intact). **b** Representative ISH images of IL1B mRNA in spinal cord motoneurons on the contralateral and on the injured side, 1 day after axotomy. Sections were counterstained with haematoxylin. Dashed lines indicate grey-white matter transition. Arrows represent motoneurons with strong staining. **c** Changes in the expression of NLRP3, CASP1 and IL1B mRNAs 1 day after axotomy in animals treated with vehicle, 5-BDBD or MCC950, compared to intact animals. *N* = 3 animals/group, *p < 0.05, **p < 0.01, ***p < 0.001 (compared to intact), ^#^*p* < 0.05, ^##^*p* < 0.01, ^###^*p* < 0.001, *n.s.* non-significant (compared to ax. + vehicle) (ANOVA with Fisher’s LSD post hoc). *Ax.* axotomy. Mean values are shown on each bar. Bars represent average ± SEM

First, expression of inflammasome components was evaluated in the L4–L5 spinal cord segments (i.e., the cell bodies of injured neurons) in animals exposed to sciatic nerve axotomy. The mRNA level of NLRP3 was significantly upregulated in response to nerve injury with a peak on days 1–3 (Fig. 1a). A similar time course was seen in the upregulation of other inflammasome-forming receptors, such as AIM2 (Additional file 1: Fig. S1b) and NLRP6 (Additional file 1: Fig. S1c), and also of caspase-1 and IL1B mRNAs (Fig. 1a), with an onset of the increase already at 6 h after sciatic nerve injury, suggesting that the priming step of inflammasome activation in the injured neurons occurs just hours after the peripheral nerve axotomy. On the other hand, there was no change in the expression of the P2X4 purinergic receptor in the first 3 days after axotomy (Additional file 1: Fig. S1d). In order to identify the cell type in which IL1B mRNA expression was increased, we performed ISH studies. On day 1 after the axotomy, no IL1B mRNA was detected on the contralateral side, whereas a moderately intense staining was detected in the ventral horn of the injured side of the spinal cord. Based on the anatomical location, cell size and cytoplasmic haematoxylin staining pattern, IL1B mRNA was clearly identified almost exclusively in motoneurons (Fig. 1b).

We presumed that extracellular ATP released by injured cells and subsequent purinergic signaling—which is a powerful NLRP3 activation signal—is involved in the inflammasomal reaction in nerve injury. Therefore, animals were administered with the P2X4 purinergic receptor inhibitor 5-BDBD and expression of NLRP3, caspase-1 and IL1B genes was assessed on day 1 after the surgery, when all three genes were highly upregulated in non-treated animals. 5-BDBD significantly reduced the axotomy-dependent upregulation of all three inflammasome-related genes, close to control levels (Fig. 1c). In addition, the NLRP3 inhibitor MCC950 could also reverse the axotomy-induced increase in NLRP3 and IL1B mRNAs. A similar tendency was observed for caspase-1, but the decrease was not significant (Fig. 1c). Since ATP is principally considered an activator stimulus, rather than a priming signal, and MCC950 is a classical inhibitor of NLRP3 activation, based on these results, we presume that priming and activation may mutually trigger each other, as IL-1β is both the end-product and also a priming agent in this system.

**Fig. 2:**
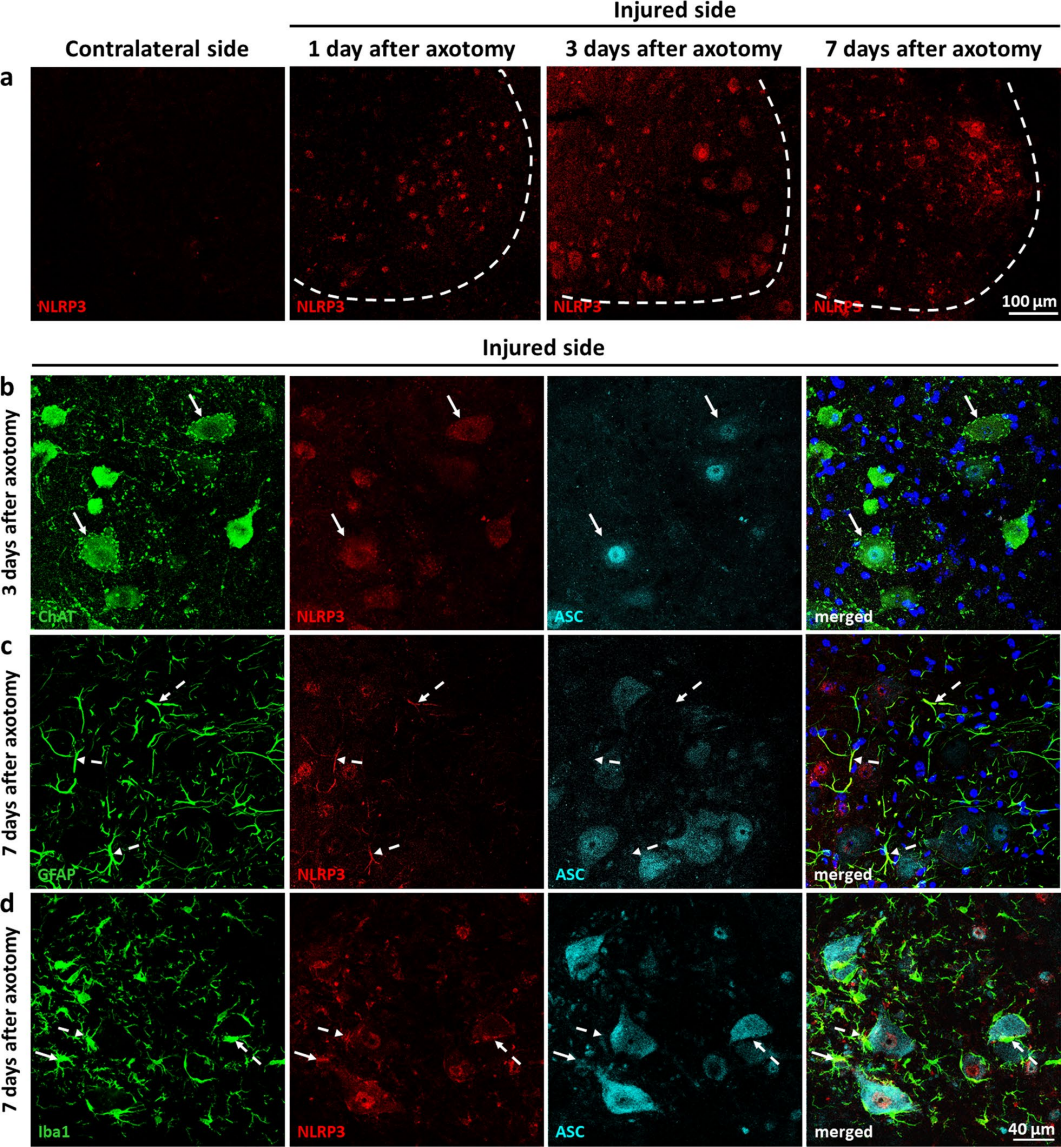
Cellular localization of inflammasome components after sciatic nerve axotomy in the spinal cord.** a** NLRP3 expression in the ventral horn of the contralateral side, and of the injured side 1, 3 and 7 days following unilateral sciatic nerve axotomy. Dashed lines represent grey-white matter transition. **b** Colocalization of NLRP3 and ASC in motoneurons labeled with ChAT 3 days following nerve injury. Arrows point at NLRP3-ASC-positive motoneurons. **c** NLRP3 expression in GFAP-positive astroglia at 7 days following axotomy. Coexpression of NLRP3 and ASC was not observed in astroglia, as represented by dashed arrows. **d** Microglial NLRP3 around injured motoneurons 7 days after axotomy. NLRP3-positive microglia were mostly ASC-negative (dashed arrows); however, NLRP3-ASC-positive microglial cells were also sparsely observed (solid arrow)

In order to clarify whether changes in gene expression were translated to the protein level, we performed IF and colocalization studies. NLRP3 protein expression was upregulated in the injured side in all time points studied (Fig. 2a). Already on days 1 and 3, NLRP3 was observed in neurons, as shown by NeuN and NLRP3 costaining (Additional file 1: Fig. S2), expressly in motoneurons, as revealed by ChAT and NLRP3 costaining (Fig. 2b). The staining was either cytoplasmic or cytoplasmic and nuclear. Notably, NLRP3 showed strong colocalization with the inflammasome adaptor protein ASC in the nuclei and later on in the cytoplasm of motoneurons (Fig. 2b; Additional file 1: Fig. S3), predicting inflammasome assembly. However, the two proteins showed partially distinct expression features, suggestive of possible inflammasome-independent functions as well. While NLRP3 was almost exclusively expressed in neurons in the first 3 days, on day 7 post-injury the NLRP3 signal appeared in GFAP- (Fig. 2c) and Iba1-positive cells (Fig. 2d) as well; however, without significant colocalization with ASC (Fig. 2c, d; Additional file 1: Fig. S4 and Additional file 1: Fig. S5). Nevertheless, NLRP3-positive microglia showed an activated phenotype, having their branches thickened and retracted, and localized in the proximity of the injured motoneurons (Fig. 2d).

**Fig. 3:**
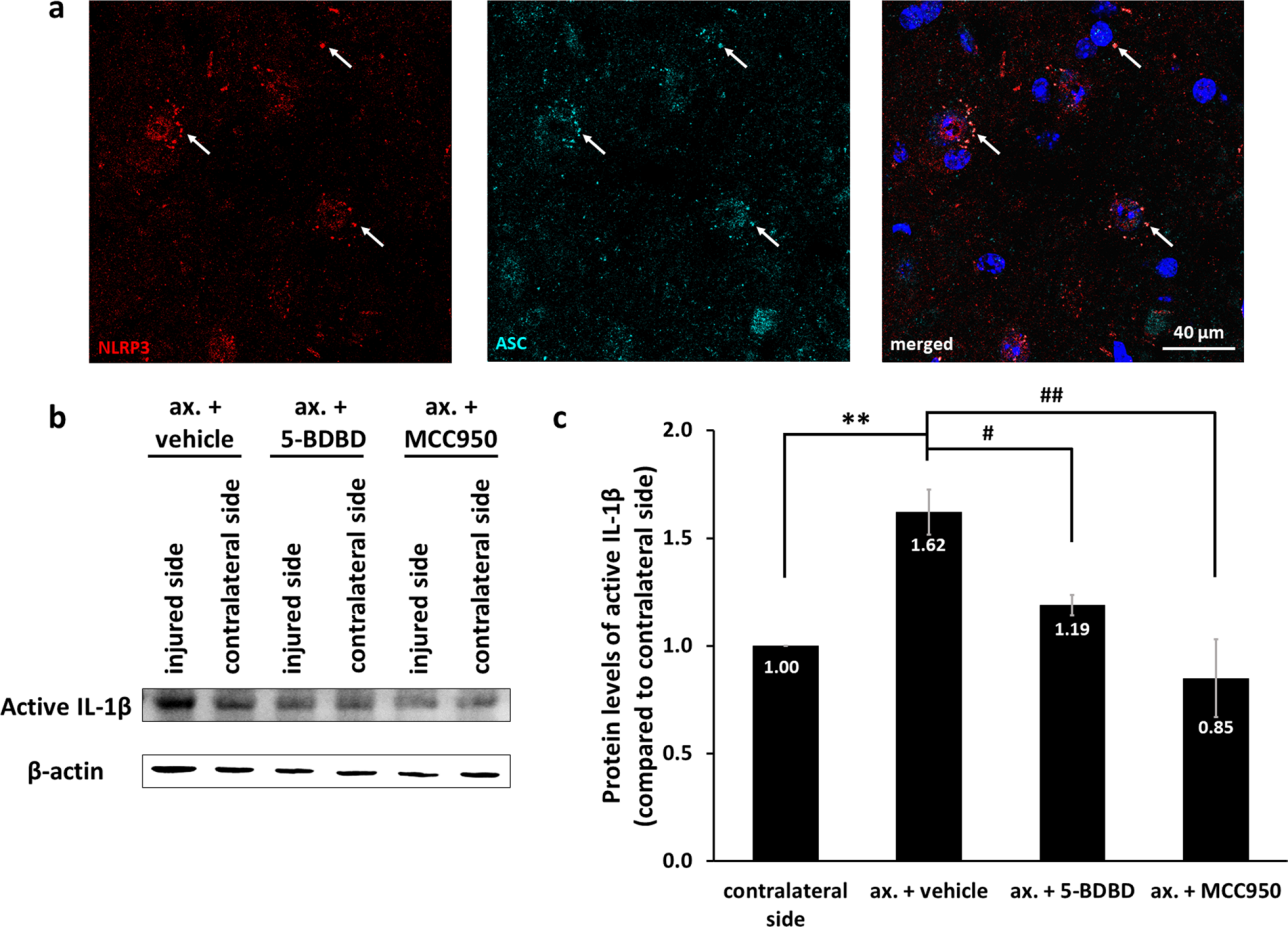
Inflammasome assembly and mature IL-1β release in the spinal cord after sciatic nerve axotomy.** a** Speck-like costaining of NLRP3 and ASC in injured neurons 3 days after the injury (arrows). **b** Representative WB images of mature IL-1β and β-actin proteins 3 days after unilateral axotomy of sciatic nerve. **c** Quantification of active IL-1β protein expression. *N* = 3 animals/group. The graph shows values normalized to β-actin levels and relative to contralateral side. Mean values are shown on bars. Bars represent average ± SEM. **p < 0.01 (ANOVA with Bonferroni post hoc, ax. + vehicle vs. contralateral side). ^#^*p* < 0.05, ^##^*p* < 0.01 (ANOVA with Bonferroni post hoc, compared to ax. + vehicle). *Ax.* axotomy

Following an increased expression of inflammasome constituents, which is the priming step, activation involves the assembly of inflammasome components. In order to monitor this step, we investigated the colocalization of NLRP3 and ASC at higher magnification to be able to observe speck formation. The speck-like costaining, as shown in Fig. 3a, indicated inflammasome activation in neurons. Inflammasome activation was further confirmed by detection of increased levels of cleaved IL-1β (i.e., the active form) in the spinal cord after nerve injury, which was inhibited by both 5-BDBD and MCC950 (Fig. 3b; Additional file 1: Fig. S6). Increase in the protein levels of IL-1β in the ipsilateral (injured) side was significant in comparison to the contralateral side, while the 5-BDBD- and MCC950-induced decrease was also significant in comparison to the vehicle-treated animals (Fig. 3c).

All these data show that in the first 3 days after the injury, the NLRP3 inflammasome is activated in the spinal cord specifically in motoneurons affected by the axotomy of the sciatic nerve.

Importantly, NLRP3 and ASC were upregulated not only in the spinal cord, but also in Schwann cells and in the microglia/macrophage population of the proximal end of the injured sciatic nerve, 3 days after the injury, accompanied by the increase of p75 and Iba1 expression as well (Additional file 1: Fig. S7 and Additional file 1: Fig. S8). Even though our study focused on the motor aspects of nerve injury, we have also assessed the dorsal root ganglia (DRG). In the injured DRG, NLRP3 and ASC expression was similar to the control side, on day 3 post-axotomy (Additional file 1: Fig. S9), indicating that inflammasome activation either does not occur or arises at a later time point in sensory neurons of the DRG.

### Neuronal inflammasome activation initiates microgliosis after axotomy

**Fig. 4:**
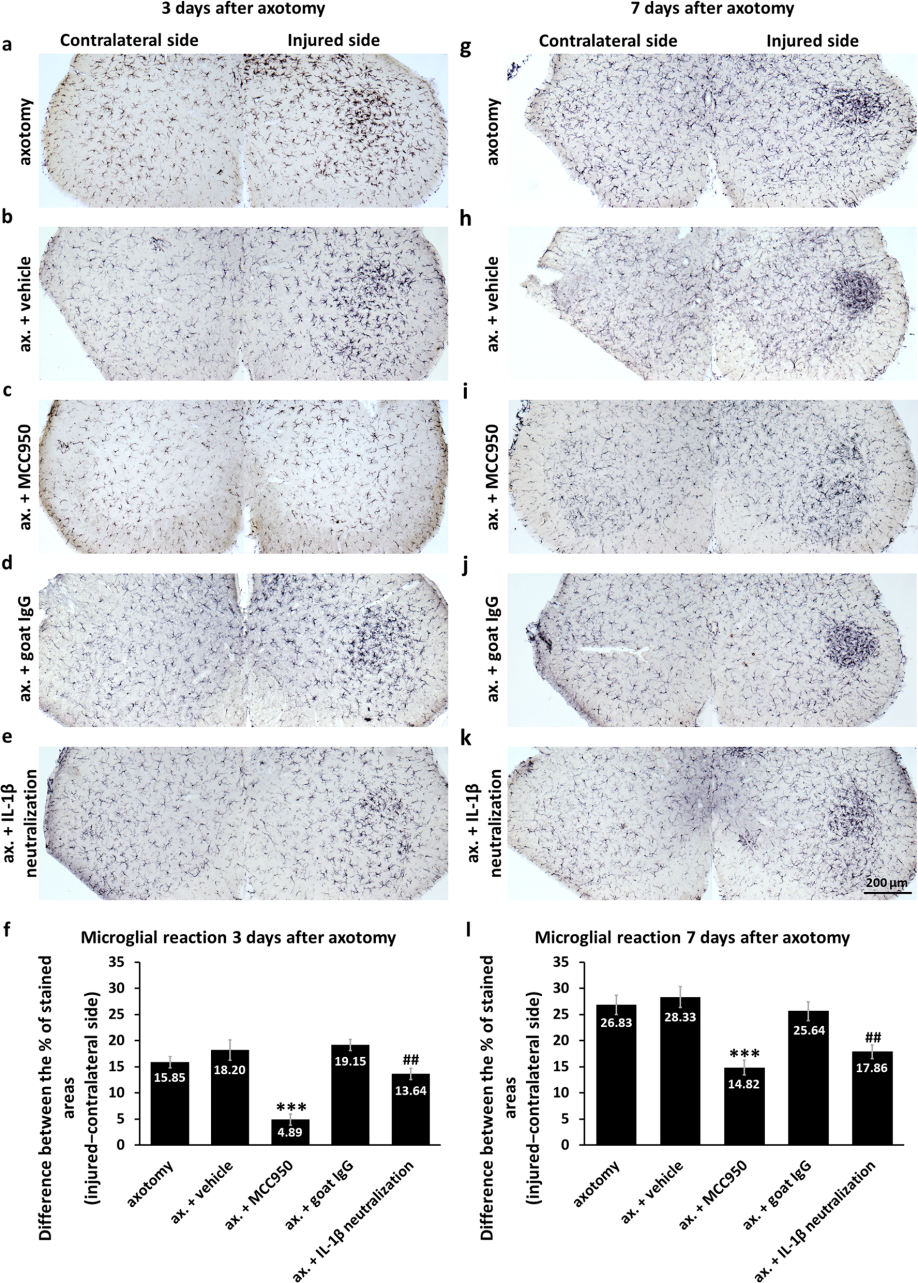
Effect of MCC950 and IL-1β neutralization on microglial activation in the spinal cord after axotomy.** a**–**e** Microglial reaction, as assessed with Iba1 IHC, in the spinal cord 3 days post-injury. **f** Quantification of microglial reaction in the ventral horn 3 days following axotomy. Results are represented as the difference between the percentage of stained area on the injured and control sides. *N* = 4 animals/group. ***p < 0.001 (ANOVA with Fisher’s LSD post hoc, compared to ax. + vehicle), ^##^*p* < 0.01 (ANOVA with Fisher’s LSD post hoc, compared to ax. + goat IgG). **g**–**k** Microglial activation in the spinal cord 7 days post-injury. **l** Quantification of microglial reaction 7 days after the axotomy. Results are represented as the difference of stained areas, similarly to (**f**). *N* = 4 animals/group. Significance marks are the same as for (**f**). Mean values are shown on all bars. Bars represent average ± SEM. *Ax.* axotomy

As a next step, assessment of the relationship between inflammasome activation and microglial activation was performed. Although faintly observable already on day 1, microgliosis became clearly detectable on the injured side on day 3 post-axotomy (Fig. 4a; Additional file 1: Fig. S10), when inflammasomal reaction was still restricted to motoneurons. Strong microglial reaction was present in both untreated (Fig. 4a) and vehicle-treated (Fig. 4b) animals, whereas MCC950 diminished the microglial reaction (Fig. 4c). Intrathecal administration of an IL-1β neutralizing antibody also reduced microgliosis compared to treatment with the goat IgG control (Fig. 4d, e). The MCC950- and IL-1β neutralization-induced reduction in microglial activation was apparent and significant (Fig. 4f).

**Fig. 5:**
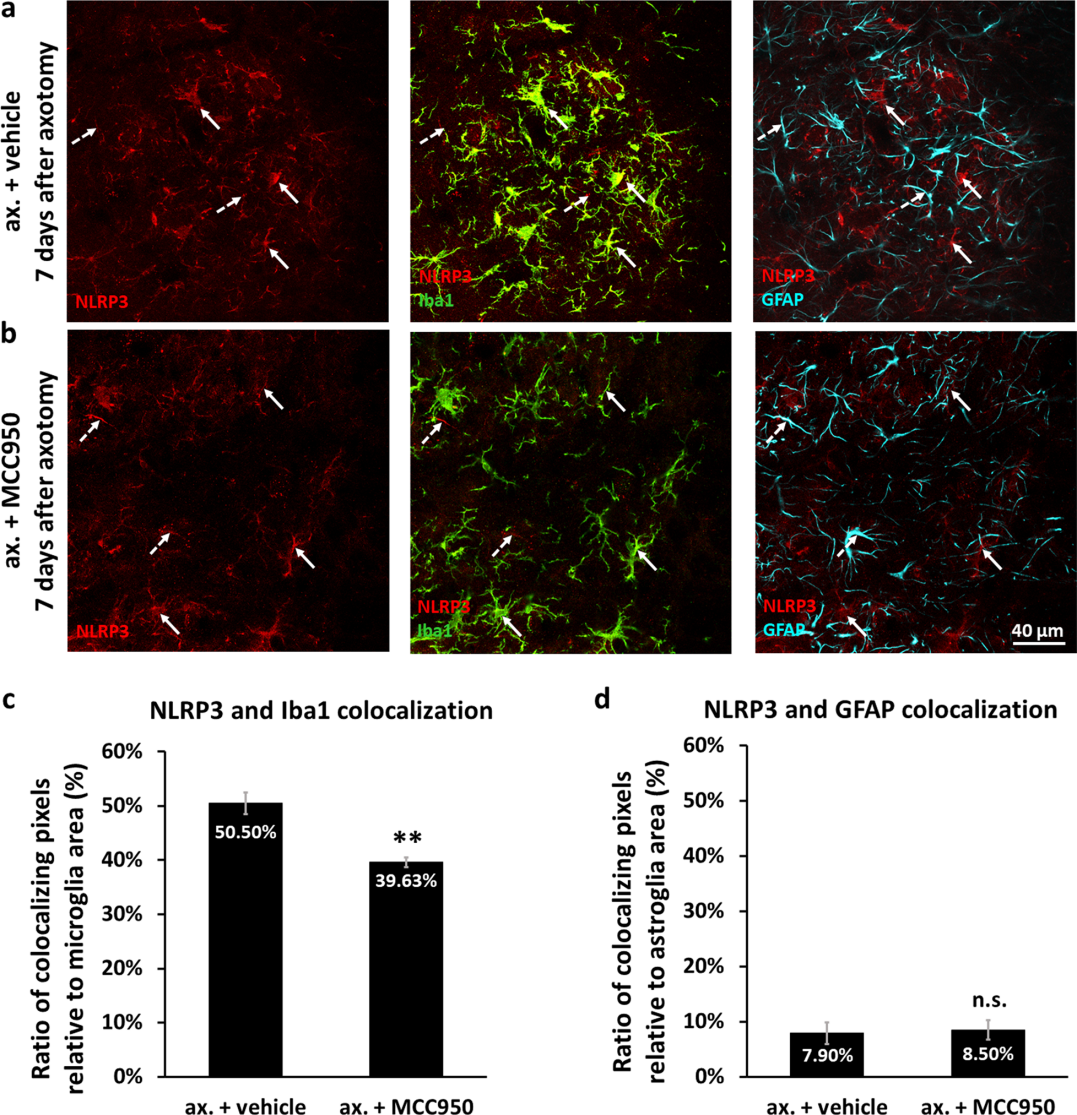
NLRP3 upregulation in glial cells 7 days after peripheral nerve axotomy. **a**, **b** Colocalization of NLRP3 with the microglial marker Iba1 (solid arrows) and with astrocytes labeled with GFAP (dashed arrows) in the injured ventral horn 7 days after sciatic nerve axotomy in vehicle- **a** and MCC950-treated animals (**b**). **c** Quantification of NLRP3-Iba1 colocalization. Results represent the ratio of colocalizing pixels, relative to microglial area. *N* = 4 animals/group, **p < 0.01 (Student’s paired t-probe). **d** Quantification of NLRP3-GFAP colocalization. Graphs indicate the ratio of colocalizing pixels, relative to astroglial area. *N* = 4 animals/group, *n.s.* non-significant (Student’s paired t-probe). Mean values are presented on all bars. Bars represent average ± SEM. *Ax.* axotomy

Microgliosis was even higher on day 7 in the injured ventral horn of untreated, vehicle-treated and IgG-treated animals; whereas administration of MCC950 or the IL-1β neutralizing antibody for 3 days (i.e., in the period when inflammasome priming and activation was present only in motoneurons) significantly decreased microglial reaction 7 days after the injury (Fig. 4g–l). Astrogliosis was much weaker than microgliosis both on days 3 and 7, and could be reduced with MCC950, but not with the IL-1β neutralizing antibody (Additional file 1: Fig. S11). In addition, administration of the NLRP3 inhibitor MCC950 in the first 3 days after axotomy, reduced the extent of colocalization of Iba1 and NLRP3 on day 7 post-surgery, but did not have any significant diminishing effect on the weak colocalization of GFAP and NLRP3 (Fig. 5; Additional file 1: Fig. S12).

These results strongly suggest that inflammasome activation in motoneurons in the first days after nerve injury is the initiator of glial reaction in the spinal cord, although a parallel cascade cannot be definitely excluded.

### Inhibition of inflammasome activation promotes neural regeneration

**Fig. 6:**
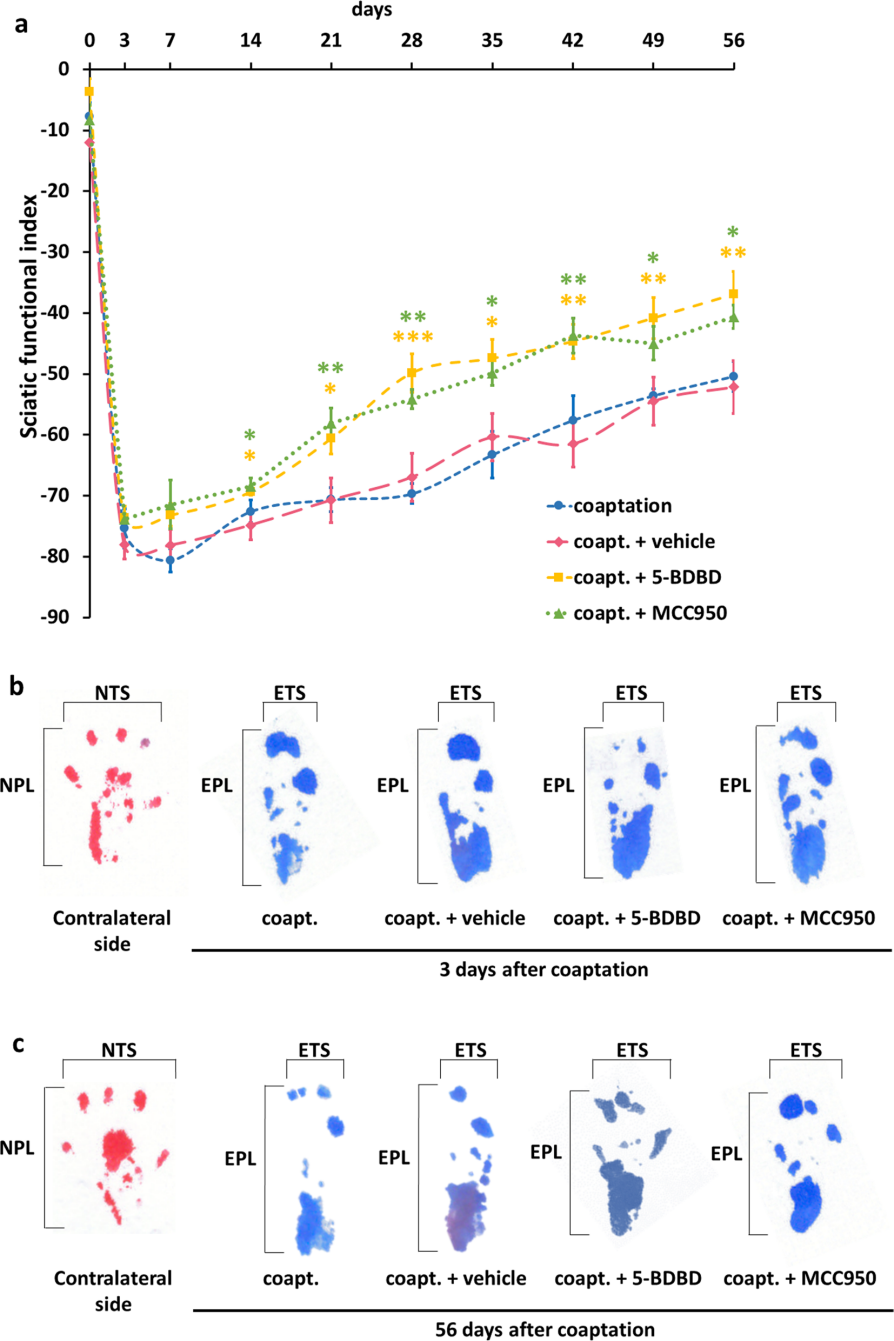
Quantification of sciatic nerve regeneration with SFI measurement. **a** SFI scores over the 8-week regeneration period following axotomy and coaptation. Dots represent SFI values at different time points, average ± SEM. *N* = 5 animals/group. *p < 0.05, **p < 0.01, ***p < 0.001 (vs. coapt. + vehicle, ANOVA with Fisher’s LSD post hoc). **b**, **c** Representative images of mouse footprints 3 days **b** and 56 days **c** after coaptation. *Coapt.* axotomy + coaptation, *NTS* normal toe spread, *NPL* normal paw length, *ETS* experimental toe spread, *EPL* experimental paw length

In order to investigate the possible functional consequences of neuronal inflammasome activation, we assessed sciatic nerve regeneration in animals whose axotomized sciatic nerve stumps were coapted with epineural sutures. Functional recovery, tested with the SFI method, was followed for 8 weeks in untreated animals and mice treated with vehicle, 5-BDBD or MCC950 in the first 3 days after axotomy and coaptation (when mainly neuronal inflammasome activation could be detected). In the untreated and vehicle-treated groups, we observed a slow, but steady increase of the SFI during the 8-week regenerative period; furthermore, our results are comparable to what other authors published using suture-based coaptation [26]. In contrast, in animals treated with 5-BDBD or MCC950, a sharp initial recovery started on day 3 after coaptation, followed by a continuous improvement of SFI values (Fig. 6). From week 2 after coaptation, a significant difference was noted between treated and untreated groups until the end of the observation period. No significant difference was detected within the treated and untreated groups, i.e., both the 5-BDBD and MCC950-treated groups performed equally well.

**Fig. 7:**
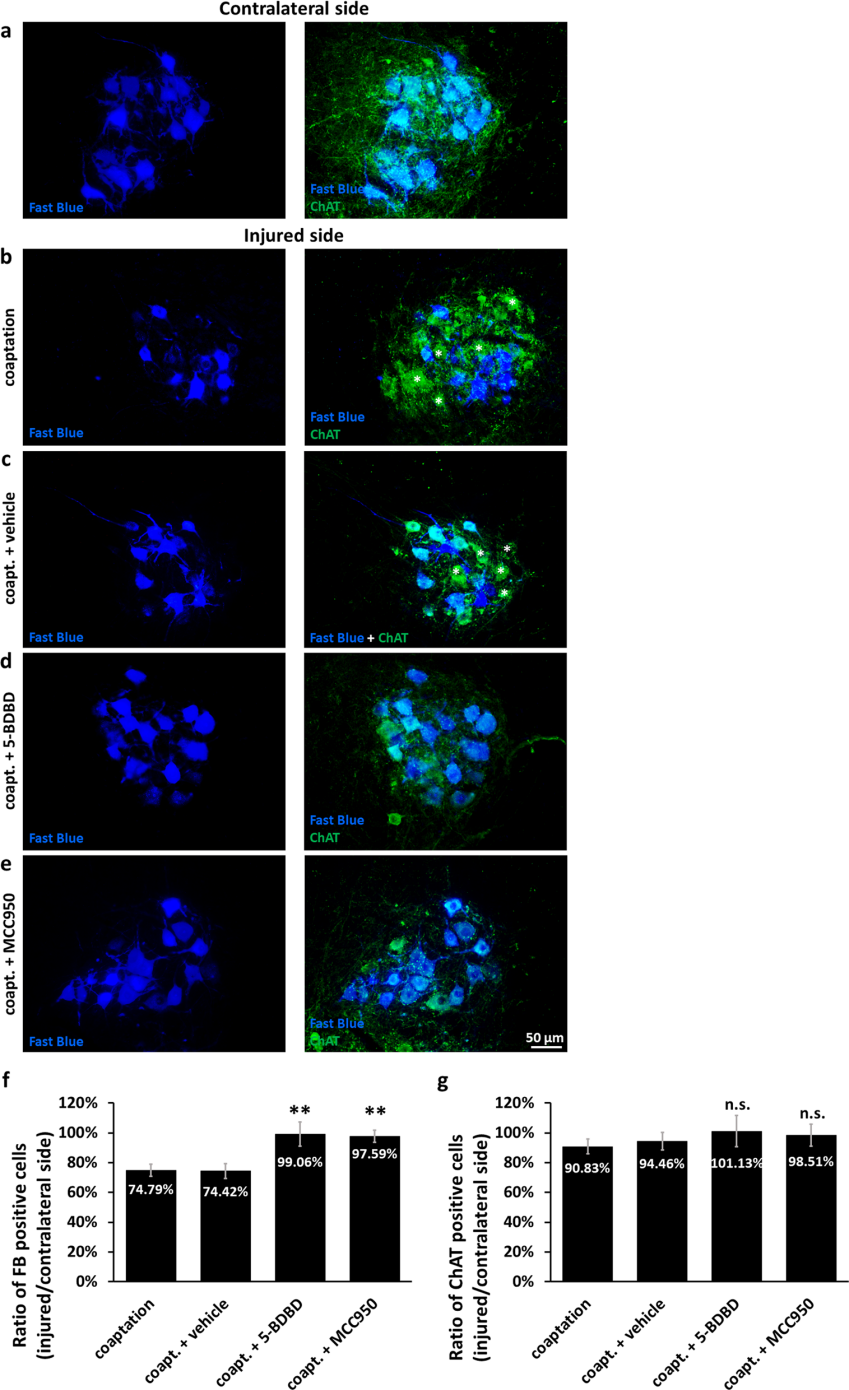
Retrograde tracing of neuronal reinnervation following sciatic nerve injury. **a**–**e** Representative images showing the FB retrograde tracer signal combined with ChAT staining in the ventral horns from the contralateral side **a** and from the injured side **b**–**e** of lumbar spinal cord, 8 weeks after axotomy and coaptation. Asterisks indicate FB-negative motoneurons. **f** Quantification of the ratio of FB-positive motoneurons (injured/contralateral side), 8 weeks after sciatic nerve axotomy + coaptation. *N* = 5 animals/group. **p < 0.01 (ANOVA with Fisher’s LSD post hoc, compared to coapt. + vehicle). **g** Quantification of the ratio of ChAT-positive motoneurons 8 weeks after sciatic nerve axotomy + coaptation. *N* = 5 animals/group. *n.s.* non-significant (ANOVA with Fisher’s LSD post hoc, compared to coapt. + vehicle). Coapt.: axotomy + coaptation. Mean values are presented on all bars. Bars represent average ± SEM

In order to quantify the number of motoneurons whose axons were able to grow through the coaptation site and regenerate into the distal sciatic nerve stump, we performed retrograde labeling of regenerating motoneurons. The sciatic nerve was cut distally from the coaptation point and FB crystals were applied for the assessment of axonal–neuronal connections based on retrograde transport of the dye (Additional file 1: Fig. S13). FB stained fewer motoneurons in the injured side of the spinal cord of untreated and vehicle-treated animals compared to the contralateral side (Fig. 7a–c), whereas 5-BDBD and MCC950 increased the number of FB-positive motoneurons in the spinal cord on the injured side (Fig. 7d, e). As expected from previous studies [27, 28], less than 80% of the total sciatic motoneuron pool were able to regenerate their axons into the distal stump in the untreated and vehicle-treated animals, while almost all axons of the whole motoneuron population grew beyond the coaptation site in animals treated with 5-BDBD or MCC950 (Fig. 7f). As expected [29], axotomy induced only a slight reduction in the number of ChAT-positive motoneurons (to 90–95%). Although the difference was not significant, both 5-BDBD and MCC950 were able to prevent motoneuron damage/loss in the injured motor pool (Fig. 7g).

**Fig. 8:**
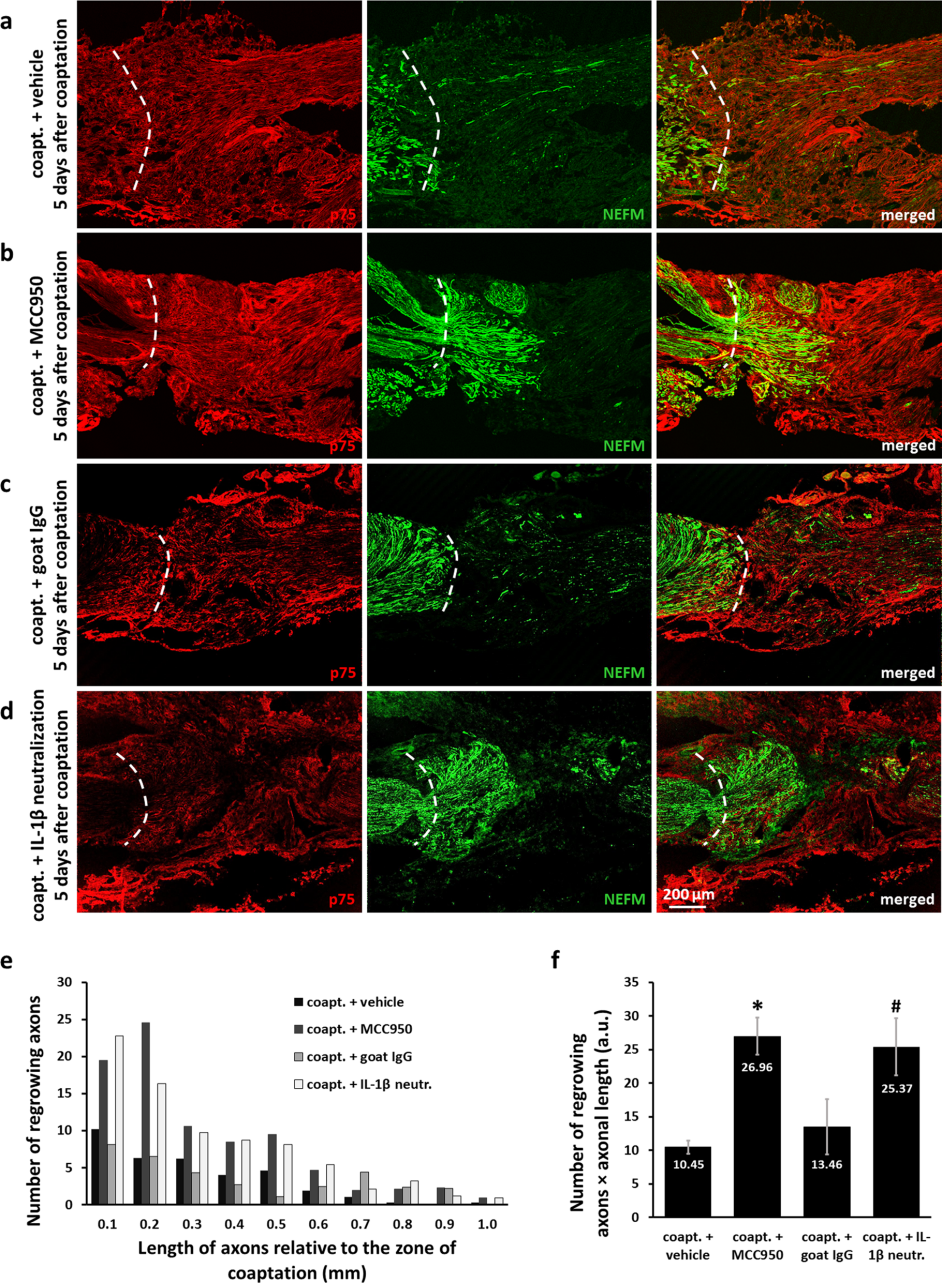
Effect of MCC950 on axonal regrowth 5 days after sciatic nerve axotomy and coaptation. **a**–**d** Confocal images of injured sciatic nerves stained with the Schwann cell marker p75 and the axonal marker NEFM. Animals were treated with vehicle (**a**), MCC950 (**b**), goat IgG **c** or IL-1β neutralizing antibody (**d**). **e** Quantification of regrowing axons 5 days after sciatic nerve injury. Bars represent the average number of regrowing axons and their distance relative to the zone of coaptation. *N* = 3–4 animals/group. **f** Graph representing the sum of regrowing axons derived as multiplication of axonal length and number of axons (i.e., area under the curve of graph (**e**)). Mean values are shown on bars. Bars represent average ± SEM. *N* = 3–4 animals/group. *p < 0.05 compared to vehicle-treated animals, ^#^*p* < 0.05 compared to mice receiving goat IgG (Student’s paired t-test). *Coapt.* axotomy + coaptation, *IL-1β neutr.* IL-1β neutralization, *a.u.* arbitrary unit

Retrograde labeling showed the extent of reinnervation at the end-point of the investigated period of time. In order to study the early events of axonal regrowth including growth rate and number of regenerating axons, we performed IF using antibodies against neurofilament (NEFM, neurofilament medium polypeptide) and the Schwann cell marker p75 in the coaptation region, 5 days after nerve axotomy and coaptation. In vehicle-treated animals, we observed very few axons growing through the coaptation zone (Fig. 8a). Treatment of the animals with MCC950 increased the length and especially the number of regenerating axons (Fig. 8b). Similar differences were observed between goat IgG-treated animals and mice receiving intrathecal IL-1β neutralization (Fig. 8c, d), underlining the role of central mechanisms. These results support our expectations that morphological improvements precede the functional changes indicated by our SFI measurements. Quantification of the number and length of regrowing axons indicated a more than twofold improvement in regeneration in the MCC950- and IL-1β neutralizing antibody-treated animals compared to vehicle- and goat IgG-treated mice, respectively (Fig. 8e, f).

These results show that inhibition of NLRP3 activation or neutralization of IL-1β in lumbar motoneurons not only hinders microgliosis but also facilitates axonal recovery after sciatic nerve injury.

## Discussion

Crosstalk of the immune and nervous systems plays a crucial role in a large number of neuropathological conditions [30, 31]. These include traumatic injuries both in the CNS and the periphery [32]; however, in peripheral nerve injury, inflammatory reactions are generally studied only locally, while examination of the central immune response – apart from a few studies [33] – is either neglected or restricted to glial cells.

Peripheral nerve injury is indeed accompanied by strong local inflammatory reactions, which have been the subject of extensive studies. These inflammatory reactions have conflicting consequences: if under control, may help in initializing regenerative processes, but over-activation can have opposite effects [34–37]. As a very potent inflammatory pathway, inflammasome activation has been identified to hinder recovery after sciatic nerve crush. In this model, NLRP3 inflammasome activation had a peak at 24 h in the injured nerve tissue, whereas absence of NLRP3 led to a faster recovery of the SFI [38]. However, another study questioned the activation of inflammasomes in sciatic nerve injury and concluded that priming of several inflammasome components is not followed by IL-1β cleavage, while NLRP6 contributes to the recovery independently of inflammasomes [39]. We observed NLRP3 upregulation and colocalization with ASC in the injured sciatic nerve, but focused our study mainly on the central mechanisms.

Injuries of spinal and peripheral nerves result in profound immunological reactions in the spinal cord. These include immune cell infiltration [40], activation of microglia [22, 33, 41] and release of immunologically active cytokines, such as IL-17, which turned out to be an important regulator of inflammatory and glial reactions following nerve injury [42]. Among pro-inflammatory cytokines, IL-1β is one of the most potent and therefore secreted in a highly regulated manner, primarily through inflammasome activation. Here we examined NLRP3 inflammasome activation and IL-1β release in the spinal cord, in the region of motoneurons of the sciatic nerve after its transection.

Quantitative PCR analysis of key inflammasome components revealed a rapid induction of these genes with a peak at 1–3 days. Surprisingly, our ISH and IF experiments identified not microglia, but motoneurons as primary sites of increased NLRP3 and IL-1β expression. Increase in the expression of inflammasome components, called priming, is the first step of inflammasome activation. In order to prove full activation of inflammasomes, we demonstrated formation of specks [43] and the presence of cleaved/active form of IL-1β. Although not primarily involved in immune reactions, neurons can express inflammasome receptors, including NLRP1, NLRC4 and AIM2 in response to different pathological conditions [44], but the role of NLRP3 in neurons has been less well investigated so far. However, besides our present findings and earlier studies [18], other results also suggest that neuronal NLRP3 can be involved in different pathologies, such as ischemic stroke [45], neurodegeneration [17, 46], epilepsy [47], trauma and sterile inflammation [48, 49]. Therefore, although microglia and astrocytes are considered the main resident cells of inflammasome activation in the CNS [50], there is increasing evidence suggesting that neurons are not only innocent bystanders in inflammatory processes and NLRP3 inflammasomes can be activated in these cells as well. Here we show that after sciatic nerve injury the main sources of active IL-1β at early stages are motoneurons in the ventral horn, suggesting that these cells can directly initiate the neuroinflammatory response.

Released IL-1β may orchestrate other inflammatory reactions, including further NLRP3 priming and microglial response as well. On day 1, when IL-1β expression already reached its maximum, we observed only a low level of microgliosis. This became evident on post-operative day 3 and—in line with observations from other laboratories [51, 52]—fully developed on day 7. At this time point, NLRP3 was already upregulated in microglia as well; however, it did not colocalize with ASC, while IL-1β expression decreased to almost control levels, suggesting absence of inflammasome assembly in these cells. Therefore, NLRP3 might have other, possibly regulatory roles in microglia in the delayed response after acute sciatic injury, as previously observed in different other cell types and models [39, 53–55]. Blockage of IL-1β in the first three days after nerve injury, significantly decreased microglial activation, suggesting that early activation of neuronal inflammasomes and neuron-derived IL-1β plays a significant role in microglial activation. Although this is the most feasible explanation, we cannot exclude the possibility, that differences in the sensitivity of the methods detecting microgliosis and NLRP3 activation, respectively, influence the time course observed by us.

Not only the consequences, but also the mediators of central inflammasome activation are interesting in the peripheral injury model. Despite the low level of motoneuronal cell death, DAMPs are released and sensed by inflammasomal or associated receptors. Extracellular ATP is one of the first mediators of tissue damage and is an important activator of the NLRP3 inflammasome through the P2X7 and P2X4 receptors [56, 57]. By inhibiting P2X4 receptors during the initial, exclusively motoneuronal inflammasome activation, we were able to reduce NLRP3 inflammasome priming and activation in the ventral horn. Moreover, administration of the P2X4 blocker in the first three days enhanced functional recovery too. Under resting conditions, although present in microglia as well [58], P2X4 receptors are mainly expressed in neurons in the spinal cord [11]. Increased P2X4 immunoreactivity was found to characterize degenerating motoneurons in the ventral horn in a transgenic rodent model of amyotrophic lateral sclerosis (ALS) [59]. In this respect, motoneuronal response to axotomy resembles ALS-like degeneration, not only through the possible role of P2X4 in neuronal injury, but also through other features, such as neuronal activation of NLRP3 [46], low level of apoptotic cell death and recruitment of microglial cells. Upregulation of P2X4 expression in hyperactive microglia, but not in astrocytes or neurons, usually occurs at later time points after nerve injury, e.g., starting from day 3 and peaking on day 14 in the spinal dorsal horn following transection of the L5 spinal nerve [60]. In line with this, we observed no P2X4 upregulation in the first three days in our model, supporting involvement neuronal P2X4 receptors in inflammasome activation.

In addition to the morphological aspects, our experiments provide evidence of the functional benefit of inflammasome blockage (i.e., axonal regrowth and sciatic reinnervation) and indicate that P2X4 or inflammasomes may serve as potential therapeutic targets in peripheral nerve injury. While in this study we hypothesize that spinal cord motoneurons are major players in inflammasome activation, the contribution of Schwann cells and microglia/macrophages to the propagation of local inflammation in the injured nerve cannot be excluded by using pharmacological inhibitors, since both 5-BDBD and MCC950—although able to penetrate the BBB and therefore target the CNS—exert their effect systemically. The role of central mechanisms is supported by literature data, indicating an opposite effect of peripheral P2X4 receptors, since overexpression of this receptor in Schwann cells was found to promote remyelination after nerve injury [61]. In addition, the importance of central vs. peripheral effects is indicated by the fact that intrathecal administration of an IL-1β neutralizing antibody had a similar beneficial effect on axonal regeneration as systemically administered MCC950.

## Conclusions

Here we provide evidence that transection of motoneuron axons distant from their cell body initiates a so far not well-understood inflammatory process both within injured axons/peripheral nerve and motoneuronal somata. This process includes activation of the NLRP3 inflammasome in motoneurons and consequent early release of the pro-inflammatory cytokine IL-1β. These changes are followed by well-characterized microglial and astroglial activation processes, leading to impaired regeneration both in the central and peripheral components of the affected motoneurons. Preventing the development of neuronal inflammation by using IL-1β neutralization, NLRP3 inflammasome blockage or a P2X4 receptor inhibitor results in improved motoneuronal survival and motor axon regeneration after peripheral nerve coaptation. Therefore, inflammasome activation in the motor neurons is rather detrimental to the recovery process and further studies are needed to understand its possible relevance as a defence mechanism.

## Supplementary Information

**Additional file 1: Fig. S1.** Gene expression changes in the spinal cord following sciatic nerve axotomy. **a** Positive control for IL1B ISH (1 day after intraspinal LPS + MDP treatment). **b**–**d** Changes in the expression of AIM2 (**b**), NLRP6 **c** and P2X4 **d** mRNAs at various time points (6 h, 1 day, 3 days, 7 days and 21 days) after sciatic nerve axotomy. Mean values are shown on each bar. Bars represent average ± SEM, N = 3 animals/group. *p < 0.05, **p < 0.01, ***p < 0.001 (ANOVA with Fisher’s LSD post hoc, compared to intact). **Fig. S2.** Localization of inflammasome components in neurons after axotomy in the spinal cord. **a**–**c** Costaining of the neuronal marker NeuN and inflammasome components NLRP3 and ASC in the ventral horn 1 day (**a**), 3 days **b** and 7 days **c** after nerve injury. Arrows indicate coexpression of all three proteins. **Fig. S3.** Localization of inflammasome components in motoneurons after axotomy in the spinal cord. **a**–**c** Costaining of motoneuronal marker ChAT and inflammasome components NLRP3 and ASC in the ventral horn 1 day (**a**), 3 days **b** and 7 days **c** after nerve injury. Arrows indicate coexpression of all three proteins. **Fig. S4.** Localization of inflammasome components in astroglia after axotomy in the spinal cord. **a**–**c** Costaining of the astroglial marker GFAP and inflammasome components NLRP3 and ASC in the ventral horn 1 day (**a**), 3 days **b** and 7 days **c** after nerve injury. Dashed arrows indicate NLRP3 expression in astroglial cells in the absence of ASC signal. **Fig. S5**. Localization of inflammasome components in microglia after axotomy in the spinal cord. **a**–**c** Costaining of the microglial marker Iba1 and inflammasome components NLRP3 and ASC in the injured ventral horn 1 day (**a**), 3 days **b** and 7 days **c** after nerve injury. Dashed arrows indicate NLRP3 expression in microglial cells in the absence of ASC signal. NLRP3-ASC colocalization in microglia is indicated by solid arrows. **Fig. S6.** Inflammasome assembly and mature IL-1β release in spinal cord in response to sciatic nerve injury. **a**, **b** Uncropped blots of Fig. 3b. **Fig. S7.** NLRP3 and ASC expression in Schwann cells in the sciatic nerve. **a** Intact sciatic nerve from the contralateral side. **b** Proximal end of the injured sciatic nerve 3 days post-axotomy. Asterisks indicate the point of transection. **c** Colocalization of NLRP3 and ASC in Schwann cells is indicated by solid arrows. **Fig. S8.** NLRP3 and ASC expression in microglia/macrophages in the sciatic nerve. **a** Intact sciatic nerve from the contralateral side. **b** Proximal end of the injured sciatic nerve 3 days post-axotomy. Asterisks indicate the point of transection. **c** Colocalization of NLRP3 and ASC in microglia/macrophages is indicated by solid arrows. **Fig. S9.** NLRP3 and ASC expression in TRPV1-positive neurons in the DRG. **a** Expression of NLRP3 and ASC in the intact DRG from the contralateral side. **b** NLRP3 and ASC expression in TRPV1-positive neurons in the injured DRG on day 3 post-axotomy. **c** Higher magnification of NLRP3 and ASC expression in TRPV1-positive neurons. **Fig. S10.** Microglial reaction in the ventral horn after sciatic nerve axotomy. **a**–**d** Microglial staining on the contralateral side **a** and on the injured side 1 (**b**), 3 **c** and 7 days **d** after axotomy. **Fig. S11.** Quantification of astrogliosis in the spinal cord after sciatic nerve axotomy. **a**, **b** Astroglial reaction in the spinal cord on day 3 **a** and day 7 **b** post-injury. Bars represent the difference of the stained areas between the injured and control sides, average ± SEM. Mean values are shown on each bar. *N* = 4 animals/group. **p < 0.01, ***p < 0.001 (ANOVA with Fisher’s LSD post hoc, ax. + vehicle vs. ax. + MCC950). Ax.: axotomy. **Fig. S12.** NLRP3-Iba1 and NLRP3-GFAP colocalization in the spinal cord. Image processing for quantifications shown in Fig. 5c, d. **a** Upper row represents the original confocal images from the ax. + vehicle group. Lower row shows the end-point of transformation into binary format. **b** Upper row represents the original confocal images from the ax. + MCC950 group. Lower row shows the end-point of image processing in the binary format. Ax.: axotomy. **Fig. S13**. Documentation of surgical procedures. **a** Image of an intact sciatic nerve from the contralateral side. **b** The sciatic nerve 5 min after axotomy followed by epineurial coaptation of the proximal and distal stumps. **c** Apparently regenerated sciatic nerve 8 weeks after axotomy + coaptation. The sciatic nerve showed no sign of atrophy. **d** On week 8, retrograde FB labeling was performed on the injured sciatic nerve distally from the coaptation point. Arrows represent the point of coaptation.

## Data Availability

The datasets used and/or analyzed during the current study are available from the corresponding author on reasonable request.

## Abbreviations

AIM2: Absent in melanoma 2
ASC: Apoptosis-associated speck-like protein containing a caspase-recruitment domain
ALS: Amyotrophic lateral sclerosis
ATP: Adenosine triphosphate
BSA: Bovine serum albumin
CNS: Central nervous system
DAB: Diaminobenzidine tetrahydrochloride
DAMP: Damage-associated molecular pattern
DMSO: Dimethyl-sulfoxide
DRG: Dorsal root ganglia
FB: Fast blue
GFAP: Glial fibrillary acidic protein
IF: Immunofluorescence
IHC: Immunohistochemistry
IL: Interleukin
IL1B/IL-1β: Interleukin-1 beta (mRNA/protein)
ISH: In situ hybridization
LPS: Lipopolysaccharide
LRR: Leucine-rich repeat
MDP: Muramyl dipeptide
NEFM: Neurofilament medium polypeptide
NLRP: NOD-, LRR and pyrin domain-containing protein
NOD: Nucleotide-binding oligomerization domain
PBS: Phosphate-buffered saline
PFA: Paraformaldehyde
PRR: Pattern recognition receptor
qPCR: Real-time polymerase chain reaction
SFI: Sciatic functional index
TBS-T: Tris-buffered saline with 0.1% Tween-20
TPBS: PBS containing 0.2% Triton X-100
WB: Western blot
