# Supplementary material for: Motoneuronal inflammasome activation triggers excessive neuroinflammation and impedes regeneration after sciatic nerve injury
Source: J Neuroinflammation. 2022 Mar 19;19:68. doi: 10.1186/s12974-022-02427-9 (PMC8934511; doi:10.1186/s12974-022-02427-9)
Supplement: Supplementary file 1 — Additional file 1: Fig. S1. Gene expression changes in the spinal cord following sciatic nerve axotomy. a Positive control for IL1B ISH (1 day after intraspinal LPS + MDP treatment). b–d Changes in the expression of AIM2 (b), NLRP6 c and P2X4 d mRNAs at various time points (6 h, 1 day, 3 days, 7 days and 21 days) after sciatic nerve axotomy. Mean values are shown on each bar. Bars represent average ± SEM, N = 3 animals/group. *p < 0.05, **p < 0.01, ***p < 0.001 (ANOVA with Fisher’s LSD post hoc, compared to intact). Fig. S2. Localization of inflammasome components in neurons after axotomy in the spinal cord. a–c Costaining of the neuronal marker NeuN and inflammasome components NLRP3 and ASC in the ventral horn 1 day (a), 3 days b and 7 days c after nerve injury. Arrows indicate coexpression of all three proteins. Fig. S3. Localization of inflammasome components in motoneurons after axotomy in the spinal cord. a–c Costaining of motoneuronal marker ChAT and inflammasome components NLRP3 and ASC in the ventral horn 1 day (a), 3 days b and 7 days c after nerve injury. Arrows indicate coexpression of all three proteins. Fig. S4. Localization of inflammasome components in astroglia after axotomy in the spinal cord. a–c Costaining of the astroglial marker GFAP and inflammasome components NLRP3 and ASC in the ventral horn 1 day (a), 3 days b and 7 days c after nerve injury. Dashed arrows indicate NLRP3 expression in astroglial cells in the absence of ASC signal. Fig. S5. Localization of inflammasome components in microglia after axotomy in the spinal cord. a–c Costaining of the microglial marker Iba1 and inflammasome components NLRP3 and ASC in the injured ventral horn 1 day (a), 3 days b and 7 days c after nerve injury. Dashed arrows indicate NLRP3 expression in microglial cells in the absence of ASC signal. NLRP3-ASC colocalization in microglia is indicated by solid arrows. Fig. S6. Inflammasome assembly and mature IL-1β release in spinal cord in response to sciatic nerv [file 12974_2022_2427_MOESM1_ESM.pdf]

ADDITIONAL FILES

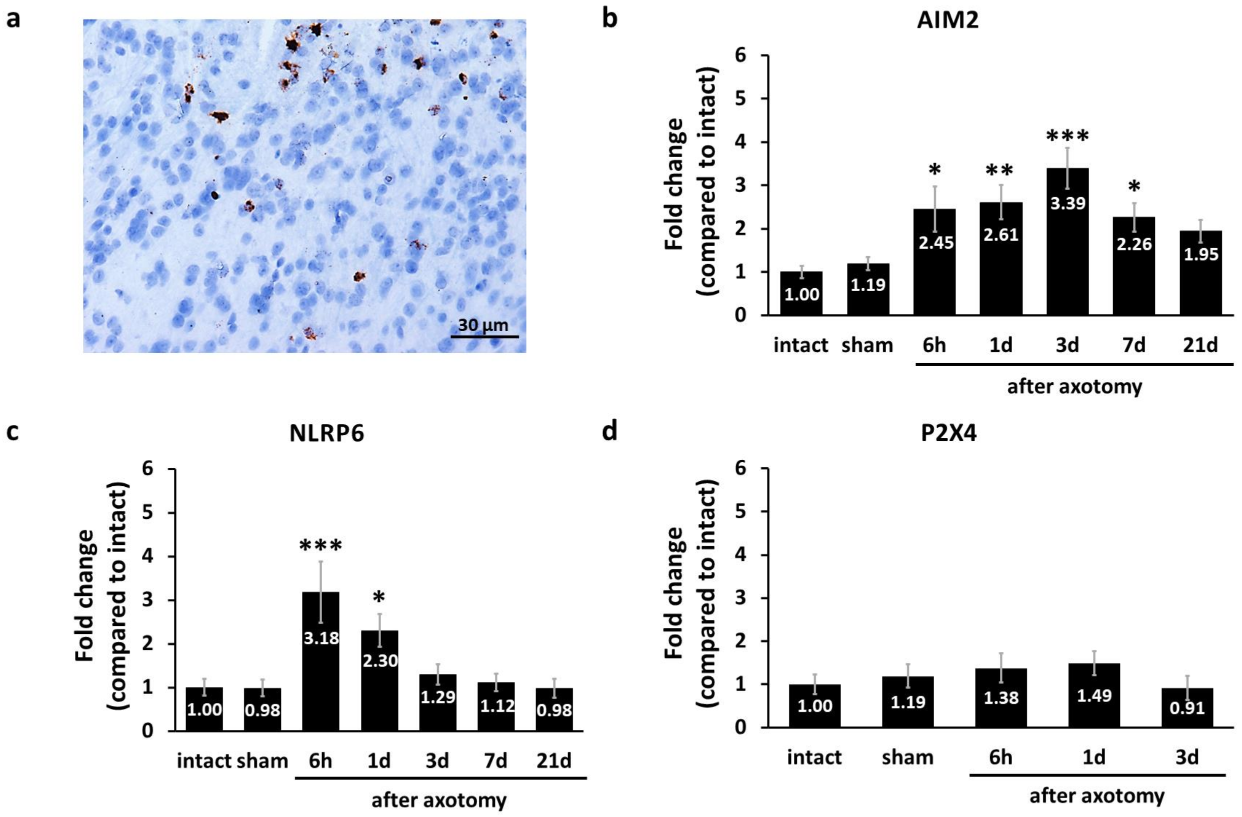

Fig. S1. Gene expression changes in the spinal cord following sciatic nerve axotomy. a Positive control for IL1B ISH (1 day after intraspinal LPS + MDP treatment). **b-d** Changes in the expression of AIM2 (**b**), NLRP6 (**c**) and P2X4 (**d**) mRNAs at various time points (6 hours, 1 day, 3 days, 7 days and 21 days) after sciatic nerve axotomy. Mean values are shown on each bar. Bars represent average ± SEM, *N* = 3 animals/group. * $p < 0.05$, ** $p < 0.01$, *** $p < 0.001$ (ANOVA with Fisher's LSD post hoc, compared to intact).

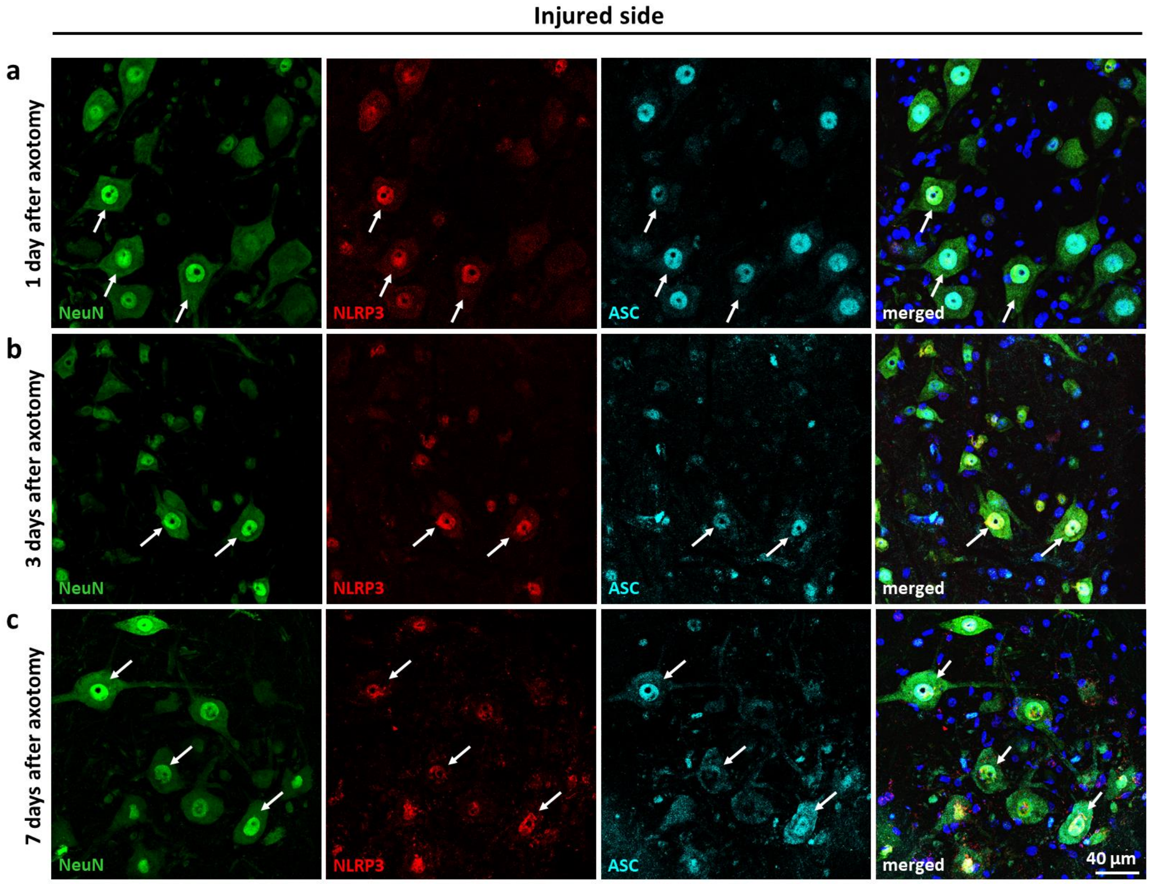

Fig. S2. Localization of inflammasome components in neurons after axotomy in the spinal cord. a-c Costaining of the neuronal marker NeuN and inflammasome components NLRP3 and ASC in the ventral horn 1 day (**a**), 3 days (**b**) and 7 days (**c**) after nerve injury. Arrows indicate coexpression of all three proteins.

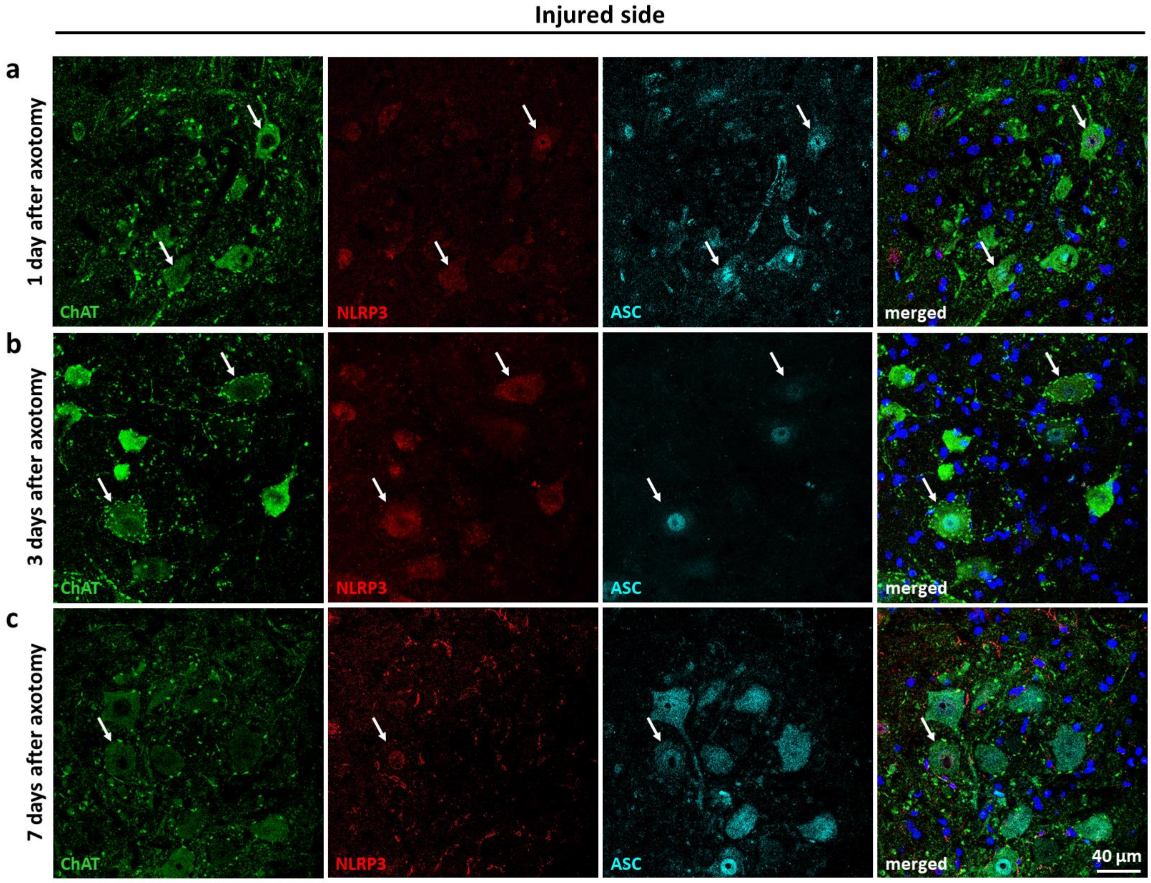

Fig. S3. Localization of inflammasome components in motoneurons after axotomy in the spinal cord. **a-c** Costaining of motoneuronal marker ChAT and inflammasome components NLRP3 and ASC in the ventral horn 1 day (**a**), 3 days (**b**) and 7 days (**c**) after nerve injury. Arrows indicate coexpression of all three proteins.

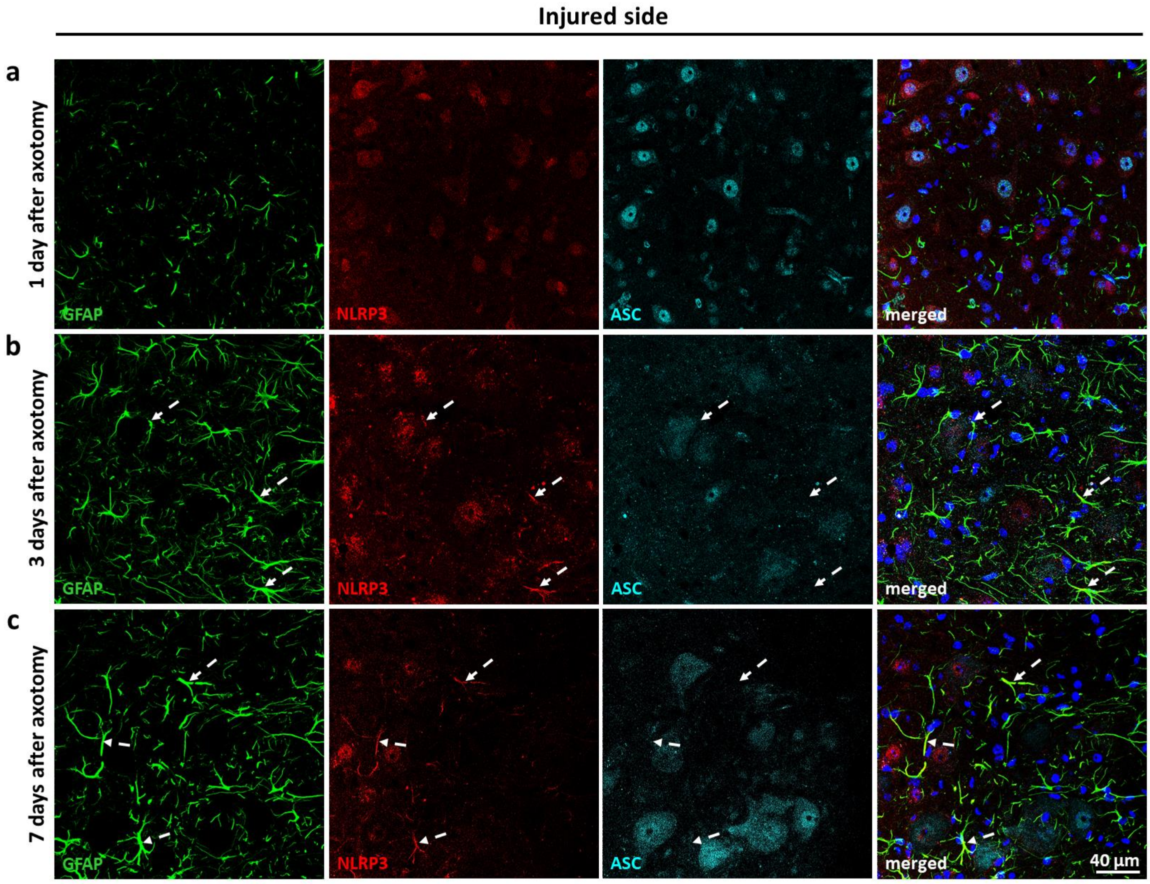

Fig. S4. Localization of inflammasome components in astroglia after axotomy in the spinal cord. a-c Costaining of the astroglial marker GFAP and inflammasome components NLRP3 and ASC in the ventral horn 1 day (**a**), 3 days (**b**) and 7 days (**c**) after nerve injury. Dashed arrows indicate NLRP3 expression in astroglial cells in the absence of ASC signal.

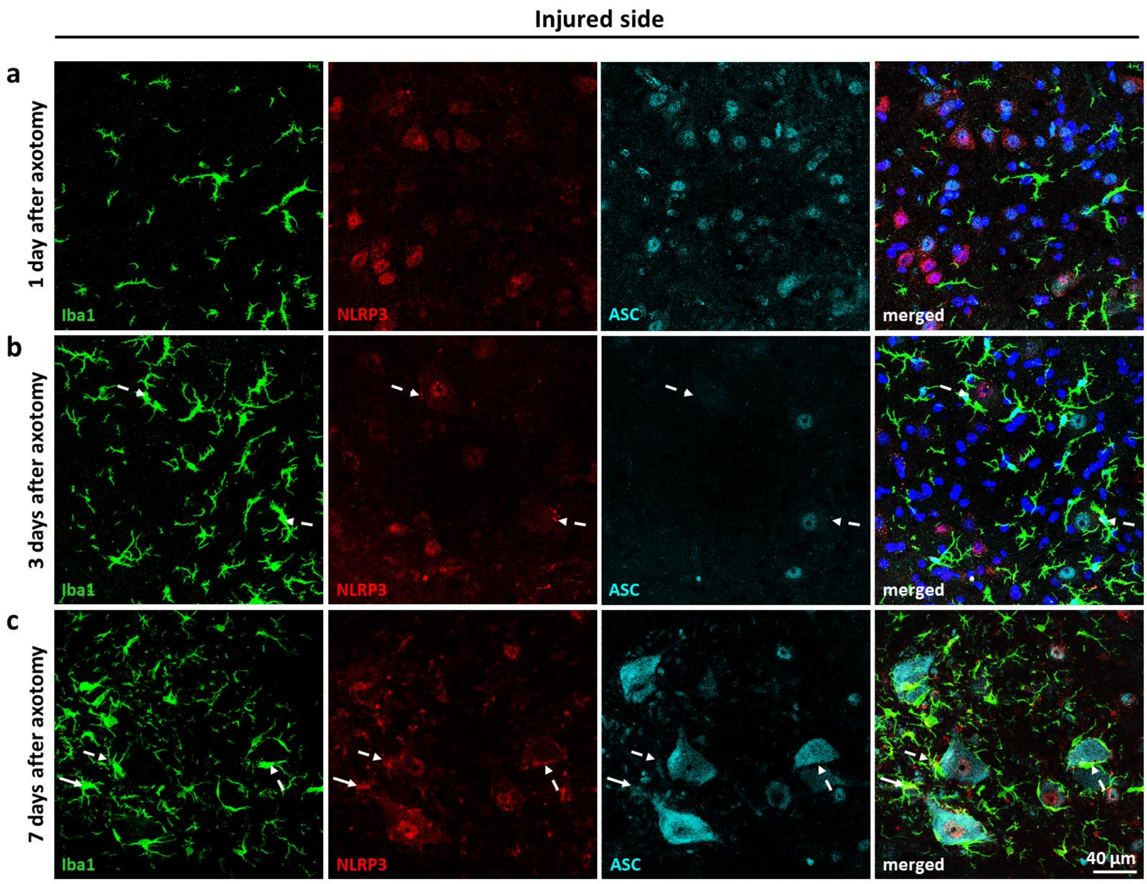

Fig. S5. Localization of inflammasome components in microglia after axotomy in the spinal cord. a-c Costaining of the microglial marker Iba1 and inflammasome components NLRP3 and ASC in the injured ventral horn 1 day (**a**), 3 days (**b**) and 7 days (**c**) after nerve injury. Dashed arrows indicate NLRP3 expression in microglial cells in the absence of ASC signal. NLRP3-ASC colocalization in microglia is indicated by solid arrows.

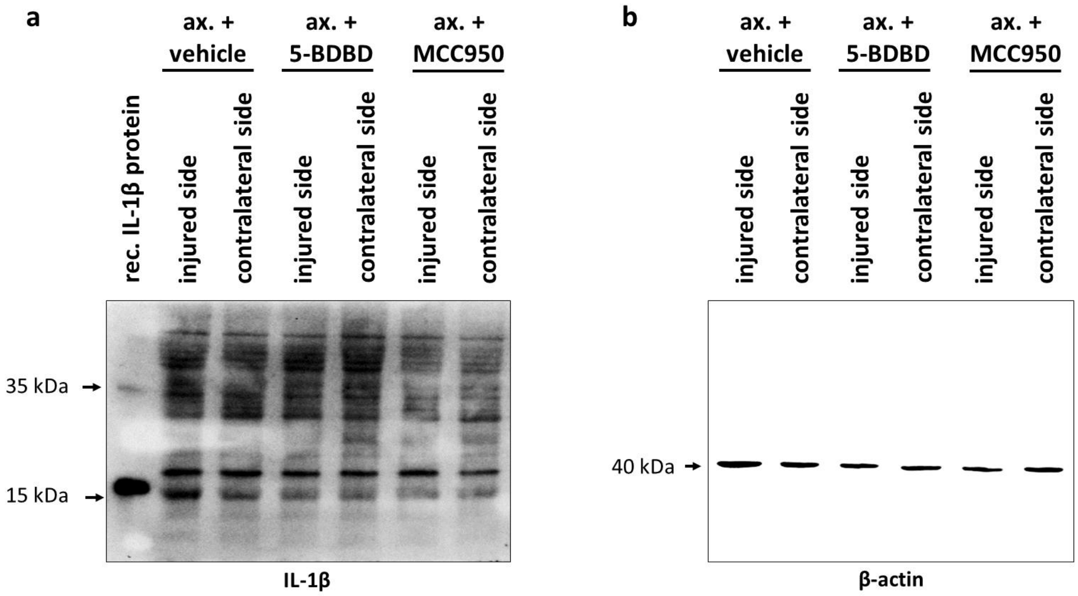

Fig. S6. Inflammasome assembly and mature IL-1β release in spinal cord in response to sciatic nerve injury. a, b Uncropped blots of Fig. **3**b.

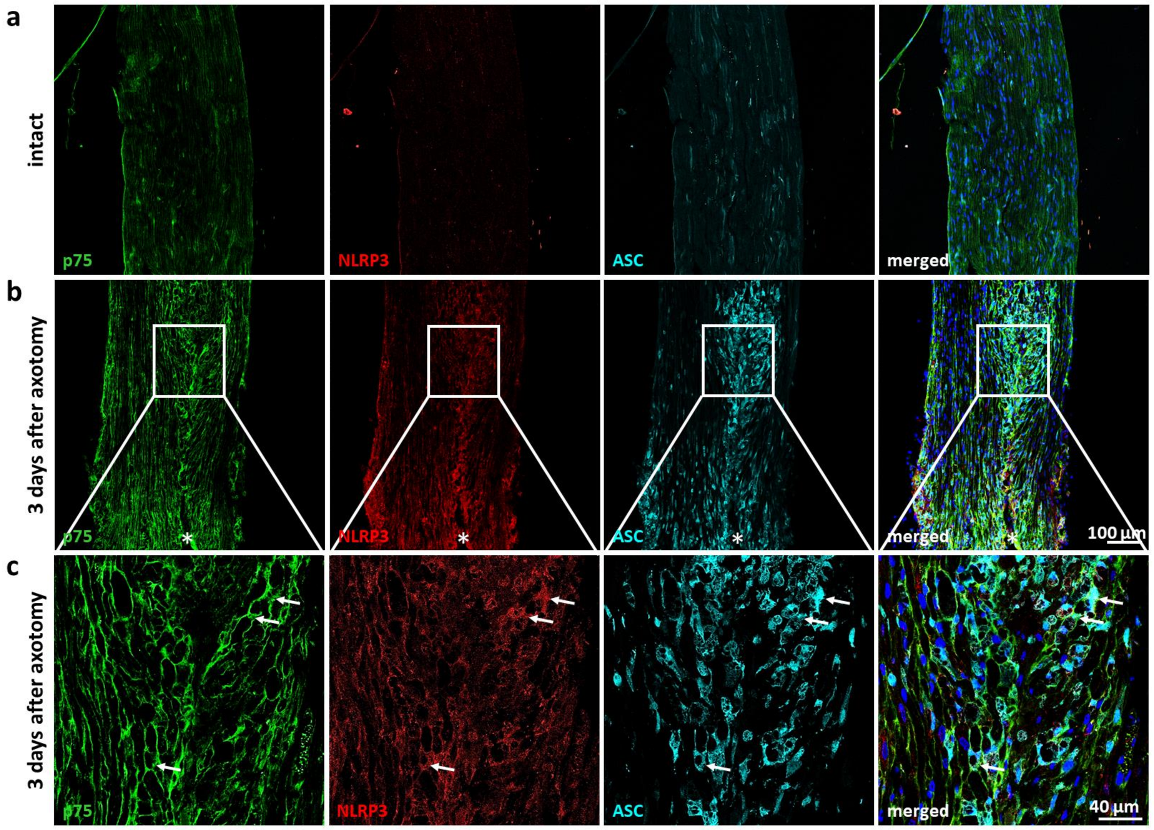

Fig. S7. NLRP3 and ASC expression in Schwann cells in the sciatic nerve. a Intact sciatic nerve from the contralateral side. **b** Proximal end of the injured sciatic nerve 3 days post-axotomy. Asterisks indicate the point of transection. **c** Colocalization of NLRP3 and ASC in Schwann cells is indicated by solid arrows.

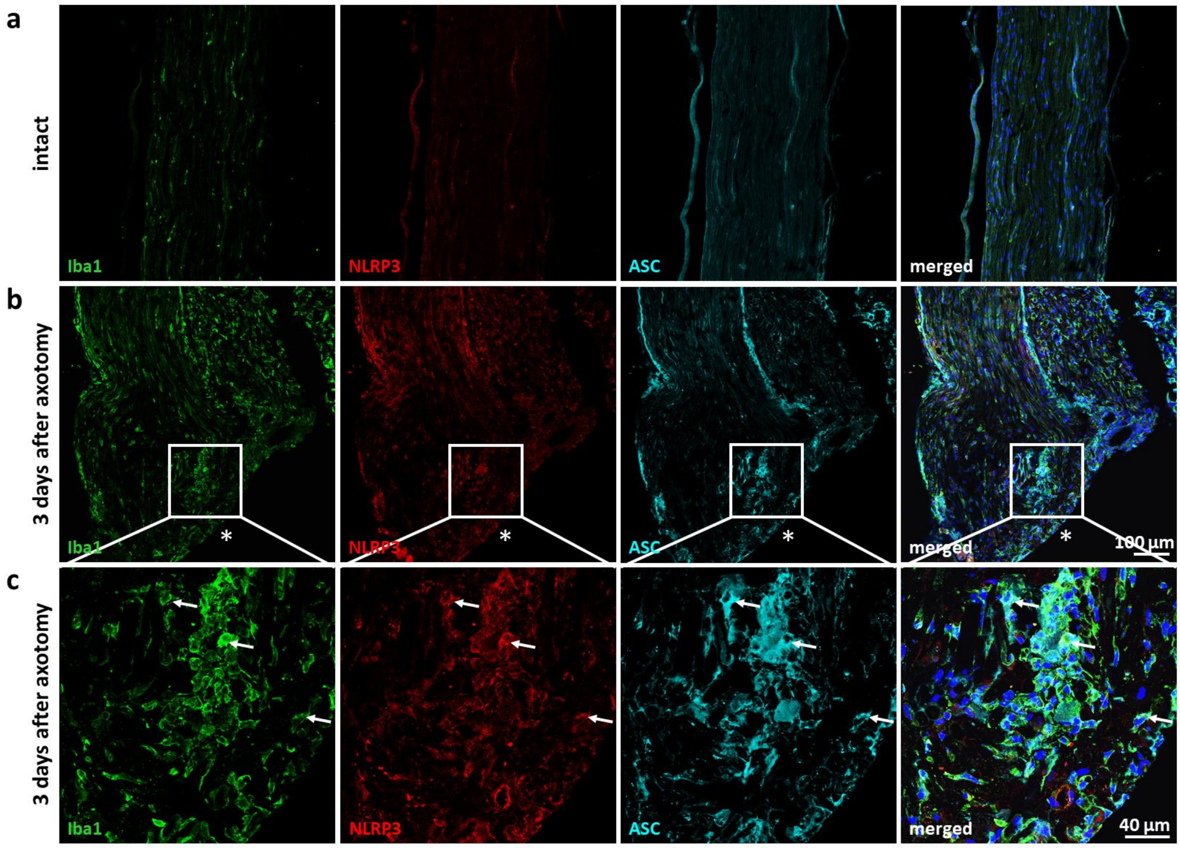

Fig. S8. NLRP3 and ASC expression in microglia/macrophages in the sciatic nerve. a Intact sciatic nerve from the contralateral side. **b** Proximal end of the injured sciatic nerve 3 days post-axotomy. Asterisks indicate the point of transection. **c** Colocalization of NLRP3 and ASC in microglia/macrophages is indicated by solid arrows.

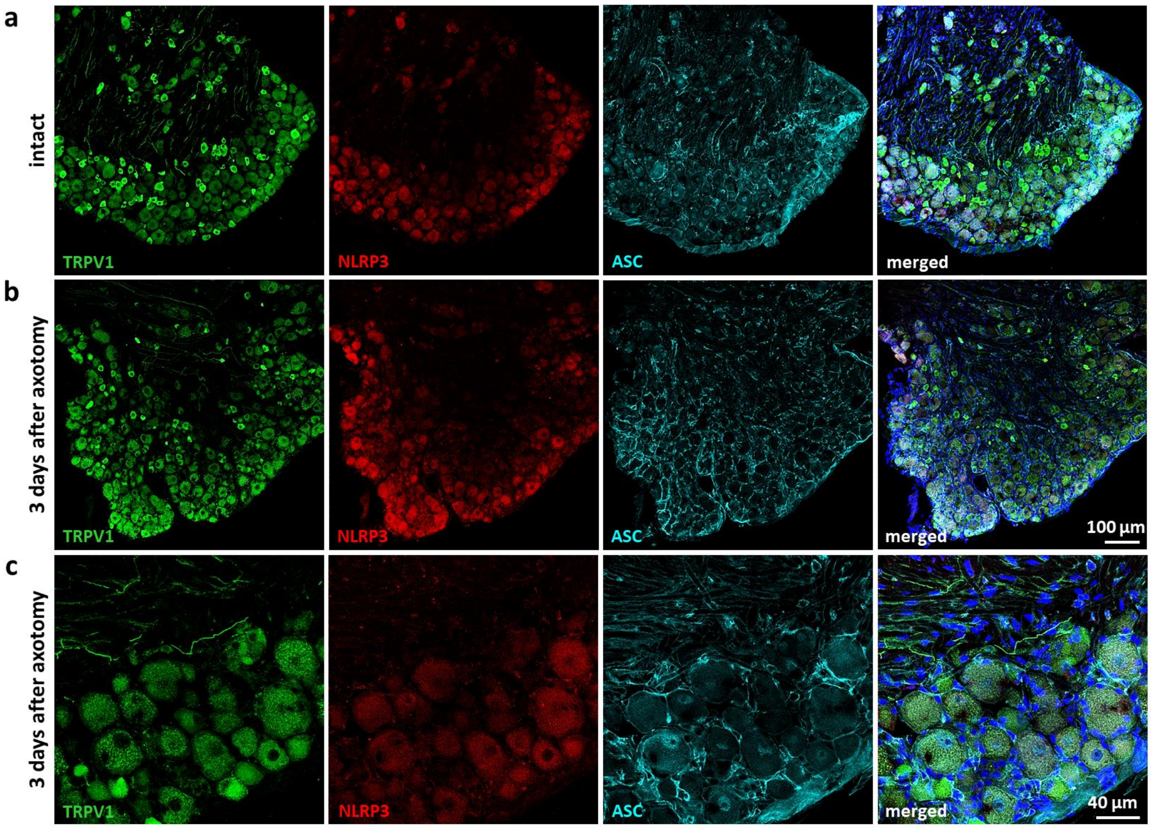

Fig. S9. NLRP3 and ASC expression in TRPV1-positive neurons in the DRG. a Expression of NLRP3 and ASC in the intact DRG from the contralateral side. **b** NLRP3 and ASC expression in TRPV1-positive neurons in the injured DRG on day 3 post-axotomy. **c** Higher magnification of NLRP3 and ASC expression in TRPV1-positive neurons.

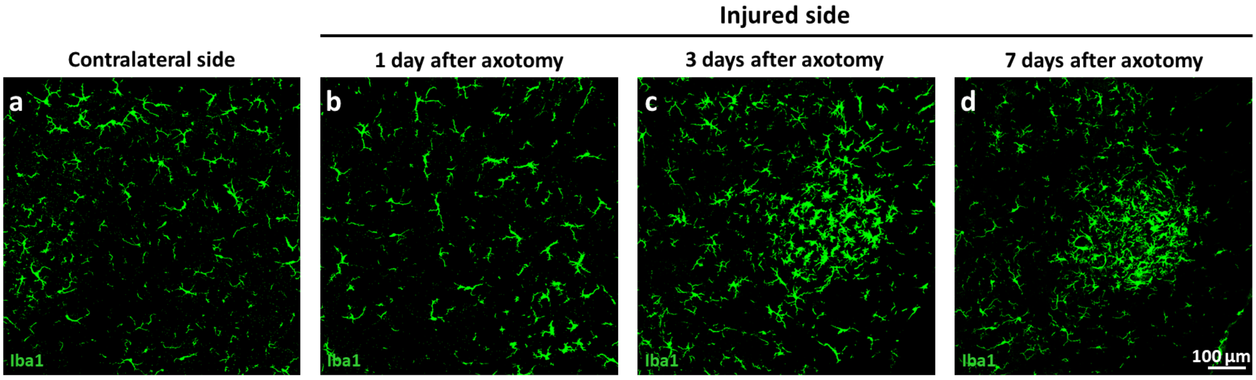

Fig. S10. Microglial reaction in the ventral horn after sciatic nerve axotomy. a-d Microglial staining on the contralateral side (**a**) and on the injured side 1 (**b**), 3 (**c**) and 7 days (**d**) after axotomy.

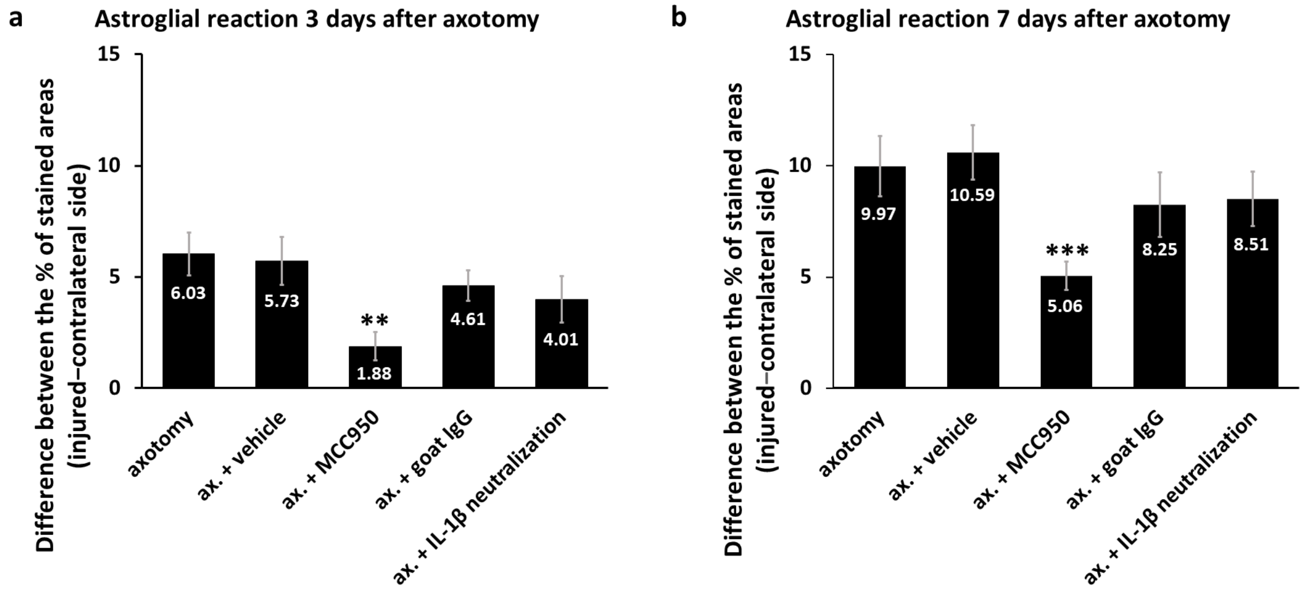

Fig. S11. Quantification of astrogliosis in the spinal cord after sciatic nerve axotomy. a, b Astroglial reaction in the spinal cord on day 3 (**a**) and day 7 (**b**) post-injury. Bars represent the difference of the stained areas between the injured and control sides, average ± SEM. Mean values are shown on each bar. *N* = 4 animals/group. ** $p < 0.01$, *** $p < 0.001$ (ANOVA with Fisher's LSD post hoc, ax. + vehicle vs. ax. + MCC950). Ax.: axotomy.

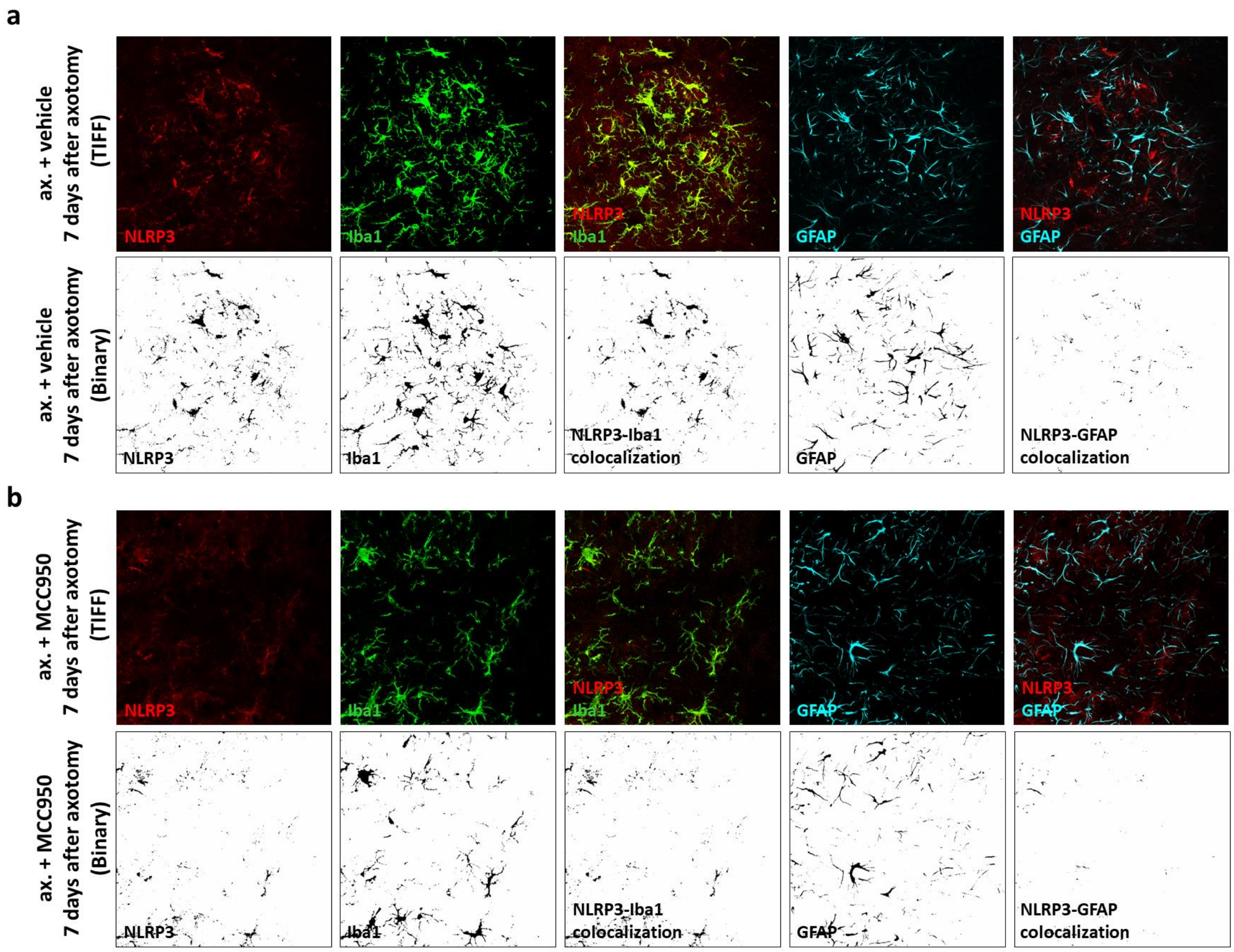

Fig. S12. NLRP3-Iba1 and NLRP3-GFAP colocalization in the spinal cord. Image processing for quantifications shown in Fig. **5**c, d. **a** Upper row represents the original confocal images from the ax. + vehicle group. Lower row shows the end-point of transformation into binary format. **b** Upper row represents the original confocal images from the ax. + MCC950 group. Lower row shows the end-point of image processing in the binary format. Ax.: axotomy.

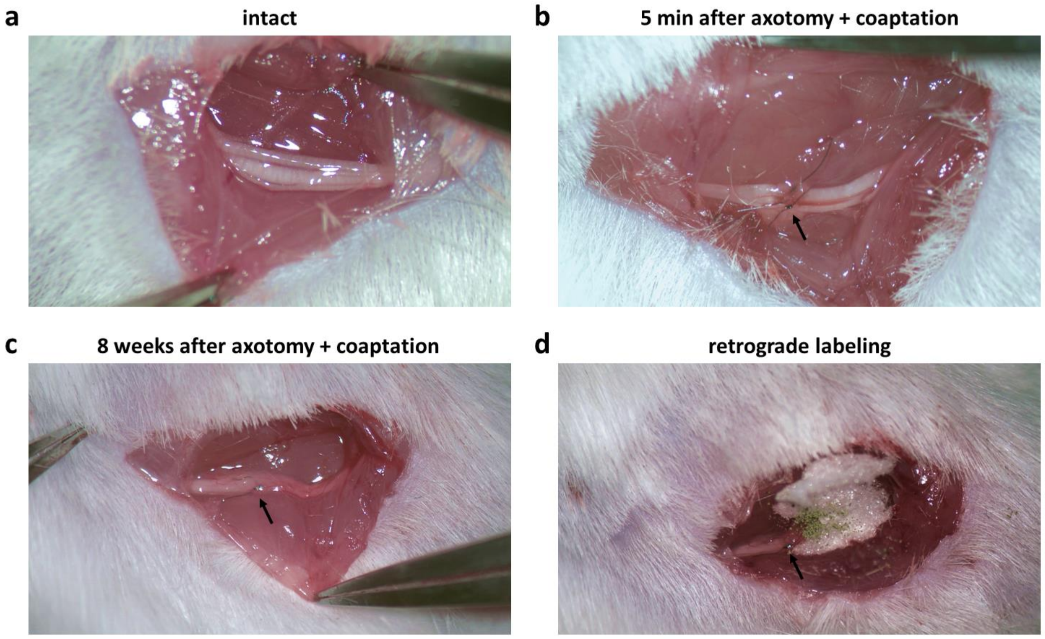

Fig. S13. Documentation of surgical procedures. a Image of an intact sciatic nerve from the contralateral side. **b** The sciatic nerve 5 min after axotomy followed by epineurial coaptation of the proximal and distal stumps. **c** Apparently regenerated sciatic nerve 8 weeks after axotomy + coaptation. The sciatic nerve showed no sign of atrophy. **d** On week 8, retrograde FB labeling was performed on the injured sciatic nerve distally from the coaptation point. Arrows represent the point of coaptation.
